# Lightning-fast genome variant detection with GROM

**DOI:** 10.1093/gigascience/gix091

**Published:** 2017-09-18

**Authors:** Sean D Smith, Joseph K Kawash, Andrey Grigoriev

**Affiliations:** Department of Biology, Center for Computational and Integrative Biology, Rutgers University, 315 Penn St, Camden 08102, NJ, USA

**Keywords:** variant detection, GROM, SNVs, structural variants, indels, copy number variants, whole genome sequencing

## Abstract

Current human whole genome sequencing projects produce massive amounts of data, often creating significant computational challenges. Different approaches have been developed for each type of genome variant and method of its detection, necessitating users to run multiple algorithms to find variants. We present Genome Rearrangement OmniMapper (GROM), a novel comprehensive variant detection algorithm accepting aligned read files as input and finding SNVs, indels, structural variants (SVs), and copy number variants (CNVs). We show that GROM outperforms state-of-the-art methods on 7 validated benchmarks using 2 whole genome sequencing (WGS) data sets. Additionally, GROM boasts lightning-fast run times, analyzing a 50× WGS human data set (NA12878) on commonly available computer hardware in 11 minutes, more than an order of magnitude (up to 72 times) faster than tools detecting a similar range of variants. Addressing the needs of big data analysis, GROM combines in 1 algorithm SNV, indel, SV, and CNV detection, providing superior speed, sensitivity, and precision. GROM is also able to detect CNVs, SNVs, and indels in non-paired-read WGS libraries, as well as SNVs and indels in whole exome or RNA sequencing data sets.

## Findings

### Introduction

The 1000 Genomes Project [1] was launched in 2008 with the goal of producing and analyzing whole genome sequencing (WGS) for 1000 genomes. By 2016 decreasing costs and increasing sequencing throughput had led to an exponential increase in the size and scope of WGS projects from Human Longevity, Inc.’s 10 000 publicly available WGS genomes [2] to the United Kingdom’s 100 000 Genomes Project [3] to even larger, though less-clearly defined, sequencing projects involving 1 000 000 participants proposed in the United States (Precision Medicine Initiative [4] and Million Veteran Program [5]) and China [6]. Such projects produce massive amounts of data, straining computational resources and requiring much faster methods than current capabilities [7].

Comprehensive analysis of genomic differences requires detection of a wide range of variants, including single nucleotide variations (SNVs), indels (insertions and deletions <50 bases), and larger copy number variants (CNVs) and structural variants (SVs), which include deletions, duplications, insertions, inversions, and translocations. Methods have been developed for each type of variant; subsequently, a typical WGS analysis workflow requires running multiple algorithms. A recent pipeline, SpeedSeq [8], focused on reducing the computational resources needed for WGS analysis, though it still employed 4 variant detection algorithms. This can be wasteful of computational resources due to repetitive input/output and analysis of the same read sequences by several algorithms.

We present our method, Genome Rearrangement Omni-Mapper (GROM), a novel comprehensive method of variant detection, combining mismatch, split-read, read pair, and read depth WGS evidence. GROM boasts lightning-speed runtimes an order of magnitude faster than state-of-the-art variant detection pipelines. While drastically reducing computational time, GROM detects SNVs, indels, SVs, and CNVs in a single algorithm and provides superior overall variant detection compared with commonly employed algorithms.

### Algorithm

**Figure 1:**
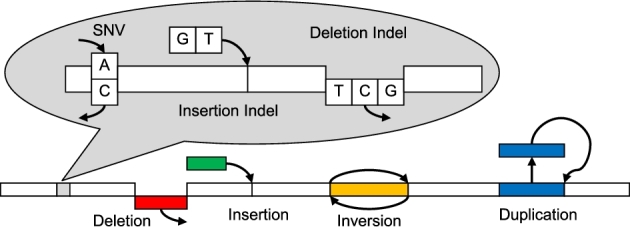
Examples of variants detected by GROM. GROM detects a comprehensive range of variants (SNVs, indels, deletions, insertions, inversions, and duplications). GROM also detects translocations spanning more than 1 chromosome (not shown).

**Figure 2:**
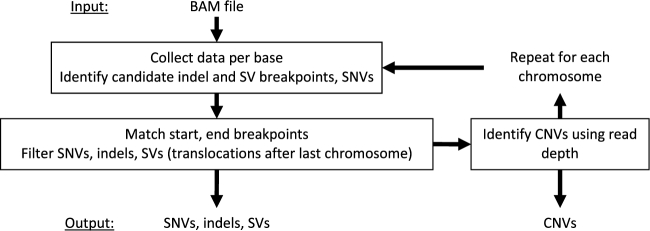
GROM workflow. GROM simultaneously collects data for each reference base and identifies candidate breakpoints and SNVs in 1 pass through a BAM file. After each chromosome, SNVs are filtered; start and end breakpoints are matched and filtered for each indel and SV type (excluding translocations), and CNVs are identified (using read depth).

**Figure 3:**
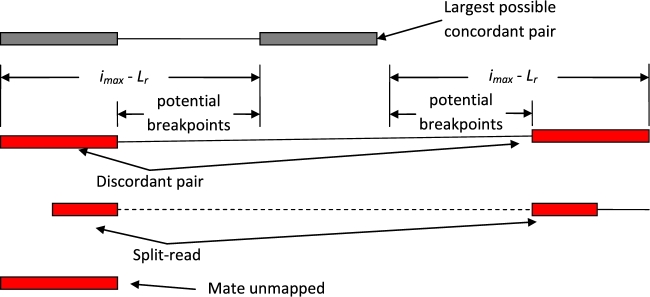
Example of SV evidence and potential breakpoints. GROM considers multiple input features at each reference base position to statistically determine the likelihood of an SNV, indel, SV, or CNV. Inputs in this example (discordant pairs, split-reads, and unmapped mate reads) are primarily used for SV detection. Discordant deletion pairs identified by insert size exceeding *i_max_*. For discordant pairs, potential start and end breakpoints are recorded for each reference base capable of forming a concordant pair in the sample. *L_r_* indicates read length.

Differences in variant types (Fig. 1) have resulted in separate algorithms designed for a limited range of variants. GROM achieves fast, comprehensive variant analysis via a compact workflow (Fig. 2), efficiently analyzing and gathering information at each reference base in 1 pass through a BAM file. Base information includes average mapping and base qualities, overlapping discordant pairs, unmapped mate reads, and split-reads, and read depth. Discordant pairs are identified based on abnormal read orientation or abnormal insert size. GROM determines abnormal insert size based on a sample of 10 million paired reads. Since insert size distributions tend to have right skewness, GROM calculates the median insert size and uses a rank-based method to determine abnormal insert size thresholds corresponding to 3 standard deviations from the median under a normal distribution (after outliers more than 5× the median insert size have been filtered). Each read with a split mapping, indel, discordant mate, or unmapped mate contributes breakpoint evidence to each potential reference base breakpoint. For simple cases such as a 2-base deletion within a read, there is 1 potential reference base start breakpoint and 1 potential reference base end breakpoint. Other cases may have less precise breakpoints, such as a read from a discordant deletion pair (abnormally large insert size). In this case, the exact breakpoint is unknown and a potential breakpoint is recorded for each reference base consistent with forming a concordant pair in the sample, where a concordant pair corresponds to insert sizes ≥*i_min_* and ≤*i_max_*, where *i_min_* and *i_max_* represent the minimum and maximum insert size thresholds, respectively (Fig. 3). Using the deletion example in Fig. 3, a breakpoint distant from both reads would necessitate an insert size that is too large to be consistent with a concordant pair (and the source DNA fragment), and thus would not be a potential breakpoint. When soft-clipping (≥5 bases) or a split-read (each mapped split ≥20 bases) occurs in the potential breakpoint region, the reference base immediately adjacent to the soft-clipping or split-read is recorded as a potential breakpoint and other potential breakpoints are recorded with half-weighting. This enables base resolution of breakpoints while limiting a single aberrant read mapping from misidentifying the true breakpoint.

Base by base of the reference, breakpoint evidence is stored for each distinct indel or SV. In some cases, it is difficult to distinguish variants. For instance, 2 heterozygous deletions may overlap and have similar start and end breakpoints and similar lengths. Thus, for each potential breakpoint, we cluster read evidence by variant type and length. Such clustering can be a computationally intensive task. We use the following efficient method.

We define a cluster or breakpoint cluster as a specific reference base location with a set of reads supporting a breakpoint at that location for a specific indel or SV type (deletion, duplication, etc.) of a certain length. A read from a discordant pair provides imprecise breakpoints and thus may be a member of multiple clusters, 1 cluster per reference location. A read is placed into an existing breakpoint cluster if the read and cluster support the same indel or SV type and the variant lengths are close, i.e.,

(1) \begin{equation*} \left| {{L_{bc}}\ -\ {L_{disc}}} \right| \le \ \left( {{i_{max}}\ - \ {i_{min}}\ + \ {i_{median}} - 2{L_r}} \right)\left( {1\ + \ \frac{1}{{{x_{bc}}}}} \right),\end{equation*}

where *L_bc_* is the mean indel or SV length for the breakpoint cluster, *L_disc_* is the length of the indel or SV pertaining to the candidate read, *L_r_* is the read length, *x_bc_* is the number of previously recorded reads supporting the breakpoint cluster, and *i_max_, i_min_*, and *i_median_* are the maximum, minimum, and median concordant pair lengths, respectively. If a candidate read does not fit in any existing breakpoint clusters, a new cluster is created. If a candidate read fits in more than 1 breakpoint cluster at the same reference position, the breakpoint cluster with the most reads is chosen. This method is efficient and has the benefit of a read being considered in multiple clusters.

Additionally, the number of previously recorded reads influences whether a read is added to a breakpoint cluster because we expect our estimated (averaged) variant length to be closer to the true SV length as supporting reads are incorporated into the SV length average. For example, in Equation (1), let insert size statistics be such that *i_max_* − *i_min_* + *i_median_* − 2*L_r_* = 500, let an SV be a deletion of 1200 bases, and let our first discordant pair indicate an SV of length *L_disc_* = 1700. One read is a poor estimate of the true SV length. Thus, in our example, the second read's SV length may differ from the first read's SV length by 1000 bases, |1700 – *L_disc_*| ≤ 1000. However, as the number of supporting reads increase, we expect the average SV length (*L_bc_*) to converge to the true SV length of 1200, at which point we will not add the read as evidence unless its estimated SV length (*L_disc_*) is within 500 bases of the true SV length, |1200 – *L_disc_*| ≤ 500*(1 + *ε*), where *ε* ≪ 1.

For each reference base, a mismapping probability, *p_bc_*, is calculated for each possible SNV, indel, and SV. *p_bc_* is the binomial probability of at least *x_bc_* reads supporting the breakpoint cluster given *n_bc_* read depth and a mapping quality threshold *m*. Thus, *p_bc_* indicates the likelihood that all of the supporting reads are mismappings. Read depth includes all mapped reads, unsequenced segments between concordant pairs, and potential breakpoints, and thus is an estimate of physical coverage. Physical coverage provides a more comprehensive representation of genome coverage than read coverage. It also helps GROM define deletion and duplication breakpoints when soft-clipping is unavailable as a decrease in coverage will affect breakpoint probability estimates. The mapping quality threshold *m* indicates the probability of a read mismapping, *p = 10^−^^m/10^*. Thus, *p_bc_* is given as

(2) \begin{equation*}{p_{bc}} = {\rm{\ Pr\ }}\left( {{\rm{X}} \ge x} \right) = {\rm{\ }}1{\rm{\ }} - {\rm{\ }}\mathop \sum \limits_{k{\rm{\ }} = {\rm{\ }}0}^{x - 1} \left( {\begin{array}{@{}*{1}{c}@{}} n\\ k \end{array}} \right){p^k}{q^{n - k}},\end{equation*}

where *q* = 1- *p*. To reduce computational time, binomial probability tables are precomputed and stored as data files. GROM will compute additional probability data files if the default mapping quality threshold (*m* = 20) is adjusted.

Potential indel and SV breakpoints are retained for further analysis. After processing reads for a chromosome (or the whole genome for translocations), GROM identifies indels and SVs with matching start and end breakpoints. Matching SV breakpoints must meet the following criteria:

(3) \begin{equation*}\left| {{B_S} + {L_S} - {B_e}} \right| \le \ c \times \left( {{i_{max}} - {i_{min}}} \right),\end{equation*}

(4) \begin{equation*}\left| {{B_e} - {L_e} - {B_s}} \right| \le c \times \left( {{i_{max}} - {i_{min}}} \right),\end{equation*}

where *c* = 3/8, *B_s_* and *B_e_* are the start and end breakpoints, respectively, and *L_s_* and *L_e_* are the average variant length of reads supporting the start or end breakpoints, respectively. Matching translocation breakpoints follow the same concept modified due to the start and end breakpoints occurring on different chromosomes,

(5) \begin{equation*}\left| {{M_S} - {B_e}} \right| \le \ c \times \left( {{i_{max}} - {i_{min}}} \right),\end{equation*}

(6) \begin{equation*}\left| {{M_e} - {B_s}} \right| \le c \times \left( {{i_{max}} - {i_{min}}} \right),\end{equation*}

where *c* = 3/8, *B_s_* and *B_e_* are the start and end breakpoints, respectively, and *M_s_* and *M_e_* are the average mate read reference locations of reads supporting the start or end breakpoints, respectively.

Mixed libraries/BAM files, e.g., with insert size distributions appreciably different as to affect Equations (3–6) for matching breakpoints, or libraries containing paired-end with mate-pair data, require separate runs of GROM. Also, GROM can analyze exome or RNA sequencing reads with detection limited to SNVs and indels.

GROM will also work for libraries of non-paired reads using (in addition to finding SNVs and SVs within reads) our earlier method for finding copy number variants (CNVs), GROM-RD [9]. GROM-RD also performs well compared with the standard tools such as CNVnator [10]. GROM and GROM-RD have the same foundation of collecting information for each reference base, but GROM-RD detects CNVs based on read depth, where low or high coverage is evidence of a deletion or duplication, respectively. This method is complementary to the core GROM approach described above.

GROM is able to simultaneously perform duplicate filtering; its duplicate filter is conceptually similar to Picard's MarkDuplicates [11] and SAMtools rmdup [12], which have been shown to have similar performance. Duplicate filtering may improve predictive accuracy relative to no filtering [13]. GROM provides an option to include such filtering, if necessary. GROM filters read pairs with identical orientation and external mapping coordinates, retaining the pair with highest mapping quality. Unlike SAMtools, GROM and Picard's MarkDuplicates are able to filter duplicates with reads mapping to different chromosomes and adjust external coordinates based on soft-clipping [13]. For the sake of speed optimization and 1-pass analysis, soft-clipping is not considered for a read's mate.

### Results

We compared GROM’s performance to 4 commonly used algorithms, GATK HaplotypeCaller (GATK-HC) [14], SAMtools [12], LUMPY [15], and Manta [16] using 2 extensively validated human WGS data sets, 51× NA12878 “platinum” genome [17] and 68× HX1, a recent Chinese genome [18]. GATK-HC, considered a gold standard in SNV/indel detection, has been shown to outperform state-of-the-art algorithms [19], and SAMtools is present in most pipelines. Because GROM integrates multiple lines of evidence, we also specifically compared it with a similar SV tool in the SpeedSeq pipeline (SpeedSeq, RRID:SCR_000469), LUMPY, shown to outperform other algorithms [15], such as DELLY (DELLY, RRID:SCR_004603) [20], Pindel (Pindel, RRID:SCR_000560) [21], and GASVPro (GASVPro, RRID:SCR_005259) [22]. As part of a 10 000 genome sequencing study, presently the largest human WGS variant study, a comparison of 7 SV detection algorithms (BreakDancer [23], DELLY [20], GenomeSTRiP [24], LUMPY [15], Manta [16], MatchClip2 [25], and Pindel [21]), showed that Manta performed the best for SV detection [2]. We evaluated SNV and indel detection with the Illumina Platinum pedigree-validated benchmark sets [17]. GROM exhibited the highest SNV and insertion indel sensitivity and precision and the highest deletion indel sensitivity when compared with GATK-HC and SAMtools for the NA12878 genome (Supplementary Table S1). SVs are notoriously difficult to reliably detect [2]. Thus, we extensively analyzed GROM’s performance using 4 benchmark sets for NA12878: Database of Genomic Variants Gold Standard (DGV-GS, deletions and duplications) [26], Mills Gold Standard (Mills-GS; deletions, duplications, and insertions) [27], Genome in a Bottle (GIAB, deletions and insertions) [28]; and Pendleton PacBio (deletions and inversions) [29]. And we utilized 3 deletion and duplication benchmark sets for HX1: DGV-GS, Shi PacBio [18], and Shi IrysChip [18] (see the Methods section for a more complete description of benchmark/validation sets). A summary of the deletion and duplication comparison with LUMPY and Manta indicated superior deletion and duplication detection (Supplementary Table S2), with GROM being the highest in 10 of 14 deletion (Supplementary Table S3) and 7 of 10 duplication (Supplemental Table S4) metrics (sensitivity and precision) across the benchmark data sets. Additionally, GROM was highest in all inversion (Supplemental Table S5) and insertion (Supplemental Table S6) metrics. GROM also detected 545 and 472 translocation events in NA12878 and HX1, respectively. However, these events were not included in the benchmarking due to the lack of validated translocation data sets for either genome.

**Figure 4:**
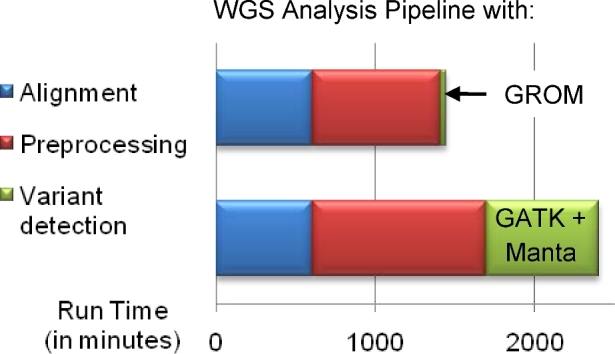
Total WGS pipeline timing on NA12878. GROM reduces WGS analysis time by drastically cutting run time for variant detection (green). It enables further speedup in preprocessing (red) by simultaneously performing an optional step, duplicate filtering. For visibility in the bar chart, GROM’s variant detection run time was artificially increased 3-fold.

With dropping sequencing costs and growing data throughput, it is imperative to reduce the computational costs of big data analysis. GROM was 1.7× (NA12878) and 2.1× (HX1) faster than the next fastest algorithm, Manta (Supplementary Table S7). Since typical analyses involve running separate algorithms for SNV/indel and SV detection, we compared a simple 24-thread parallelized GROM version (allocating a thread per 1/24 of the genome) with the fastest and best-performing 2-algorithm workflow (GATK-HC/Manta). Strikingly, GROM ranged from 24× (HX1, no duplicate filtering) to 72× (NA12878 with duplicate filtering) faster than a combination of 22-thread GATK-HC/2-thread Manta (Supplementary Table S8), drastically reducing variant detection and duplicate filtering from 41% to <1% of a typical WGS analysis pipeline (Fig. 4). For 1000 genomes on a 24-thread server, it may literally save years of computation.

Comparing the variants predicted by different tools, we identified 33 validated NA12878 SVs detected by GROM (but unreported by LUMPY and Manta) that overlapped genes and ranked them using the number of independent validations (Supplementary Table S9). A variant was considered validated if it occurred in at least 1 of the NA12878 benchmarks corresponding to the SV type (DGV-GS, Mills-GS, GIAB, Pendleton PacBio for deletions; DGV-SV, Mills-GS for duplications; Mills-GS, GIAB for insertions; and Pendleton PacBio for inversions).

**Figure 5:**
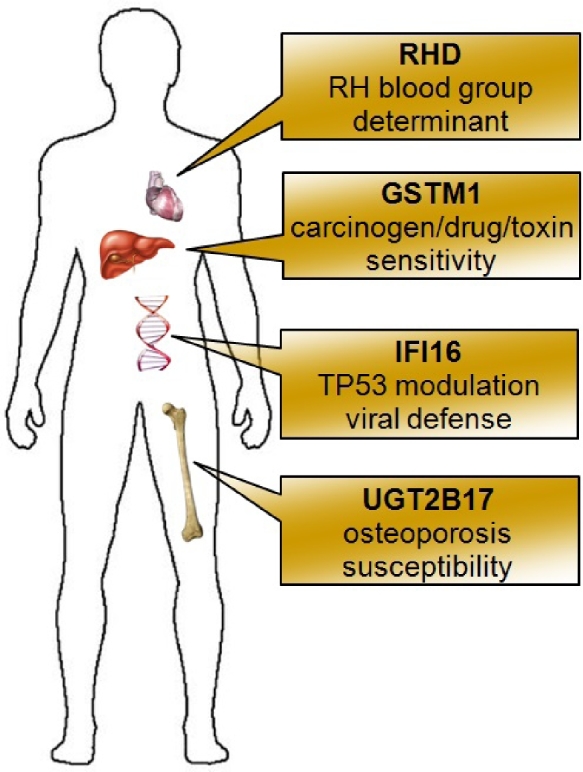
Example of genes overlapped by validated GROM-specific SVs. In the example are 4 of 33 genes overlapped by validated SVs that were identified by GROM and unreported by LUMPY and Manta. Biological significance listed below gene.

Among these variants, we noted 4 deletions with significant health-related impact for NA12878: RHD, GSTM1, IFI16, and UGT2B17 (Fig. 5). GROM predicted a deletion spanning the entire RHD gene, 1 of 2 genes responsible for Rh blood group antigens [30]. Decreased copy numbers or null genotype of GSTM1 have been associated with hepatotoxicity [31] and higher risk of many cancers including lung cancer [32], gastric cancer [33], and bladder cancer [34]. UGT2B17 copy number variation has been associated with changes in bone mineral density and risk of osteoporosis [35]. IFI16 is involved in viral defense [36] and p53-mediated apoptosis [37, 38].

Additionally, GROM provides an option to include duplicate filtering. This leads to minor accuracy gains in a number of cases (see example in Supplementary Table S10) and achieves additional speedup (Supplementary Table S8). Lastly, we have summarized GROM’s relative performance in Table 1.

**Table 1:** Comparison of GROM and leading algorithms’ variant detection accuracy and run time

		GATK-HC	SAMtools	LUMPY	Manta	GROM
SNV		2	3	-	-	**1**
Indel	Deletion	**1**	3	-	-	**1**
	Insertion	2	3	-	-	**1**
SV	Deletion	-	-	2	3	**1**
	Duplication	-	-	2	2	**1**
	Insertion	-	-	-	2	**1**
	Inversion	-	-	3	2	**1**
Run time		4	5	3	2	**1**

Performance based on sensitivity and precision rankings (1 = highest, 3 = lowest) averaged across benchmarks for NA12878 and HX1. Bold text indicates the best-performing algorithm in each category. A dash sign indicates that an algorithm does not detect variant type.

## Methods

All timings were performed on an Intel Xeon E5–2690 v. 3 processor, 2.60 GHz, with 24 threads and 128 GB RAM.

Rankings in Table 1 and Supplementary Table S2 were based on average ranking across benchmarks (1-highest to 3-lowest). Ranking for each benchmark was based on sensitivity and precision values in Supplementary Tables S3–S6. For instance, GROM had the highest value for 10, second highest for 2, and lowest for 2 of the 14 deletion sensitivity and precision benchmarks (average benchmark rank, 1.4) Subsequently, the algorithms were ranked after sorting by their average benchmark ranking, resulting in deletion rankings of GROM, 1; LUMPY, 2; and Manta, 3 (as shown in Table 1).

Unlike most SV variant callers, GROM is able to analyze data sets with single or paired reads. However, all SV tests included only paired reads since most of the other callers operate on those.

While state-of-the-art detection methods for SNVs and indels have been deemed adequate for the clinical setting, SV detection is notably more difficult [2]. Additionally, synthetic data sets have suffered from oversimplifications and misleading conclusions [2]. Thus, we extensively analyzed GROM’s SV detection performance using 4 validation benchmark sets for NA12878:

1. Database of Genomic Variants Gold Standard (deletions and duplications) [26] in Supplementary Tables S2–S4;
2. Mills Gold Standard (deletions, duplications, and insertions) [27] in Supplementary Tables S2–S5;
3. Genome in a Bottle (deletions and insertions) [28] in Supplementary Tables S2, S3, S5; and
4. Pendleton PacBio (deletions and inversions) [29] in Supplementary Tables S2, S3, S6.

Additionally, we utilized 3 deletion and duplication benchmark sets for HX1: DGV-GS (as above), Shi PacBio, and Shi IrysChip [18] in Supplementary Tables S2–S4. For NA12878 DGV-GS benchmarks, all deletions and duplications with the “NA12878” tag were extracted from the DGV-GS. The HX1 DGV-GS benchmarks were created by extracting deletions and duplications with the “Asian” tag. To obtain a benchmark set of common Asian variants, deletions and duplications with fewer than 200 “Asian”-tagged samples were filtered.

To limit potential biases, we selected benchmarks covering a range of technologies, including Illumina, PacBio, and IrysChip, and inclusive of multiple variant detection algorithms (Illumina platinum pedigree-validated, DGV-GS, Mills-GS, and GIAB). Indels were defined as deletions and insertions <50 bases, whereas SVs were ≥50 bases. To identify true positives, indel benchmarking required variant call breakpoints within 2 bases of the benchmark. Insertion SV calls within 10 bases of the benchmark were considered true. All other SV benchmarking required a 50% (10% for IrysChip due to low resolution) reciprocal overlap of a variant call and the benchmark. Some false positives may potentially be true positives not represented in the benchmark. To limit false positives due to unrepresented calls, for each SV type (excluding insertions where the length is often unknown), we ignored SV calls smaller or larger than a particular benchmark's shortest or longest SV, respectively.

NA12878 and HX1 Illumina platinum fasta files were mapped to human references hg19 and GRCh38, respectively, using BWA mem [39], version 0.7.15, with the -M parameter to mark shorter read splits as secondary. Duplicate filtering comparisons were performed using default parameters for SAMtools [12], version 1.3.1, and Sambamba [40], version 0.6.4. GATK version 3.6.0 HaplotypeCaller (GATK, RRID:SCR_001876) [14], SAMtools (SAMTOOLS, RRID:SCR_002105) [12], LUMPY (LUMPY, RRID:SCR_003253; version 0.2.11) [15], and Manta (version 1.0.1) [16] were run with default parameters.

## Conclusion

Our extensive performance analysis indicates that GROM achieves superior variant detection and is significantly faster than current state-of-the-art methods by incorporating comprehensive variant detection (SNV, indel, SV, CNV), duplicate filtering, and multi-threading in 1 algorithm. GROM’s superior variant detection makes it valuable for WGS analysis projects of all sizes, and its “lightning”-fast speed is especially critical for keeping pace with increasingly higher sequencing throughput and larger data projects.

## Availability of data and materials

NA12878 raw short-read Illumina platinum WGS data, as well as pedigree-validated SNVs and indels, supporting the results in this study are available from the Database of Genotypes and Phenotypes under accession number phs001224.v1.p1 [41]. HX1 raw short-read Illumina WGS data supporting the results in this study are available from the National Center for Biotechnology Information (NCBI) Sequence Read Archive (SRA), study PRJNA301527 [42]. DGV-GS-validated SVs supporting the results in this study are available from the Database of Genomic Variants website [43]. Mills-GS-validated SVs supporting the results in this study are available as Supplementary Table S5 in the associated paper [27]. GIAB validation data supporting the results in this study are available from NCBI at separate locations for deletions (v. 3.3.1) [44] and insertions [45]. Pendleton PacBio–validated deletions and inversions supporting the results in this study are available as Supplementary Tables S5 and S6, respectively, in the associated paper [29]. Shi PacBio– and Shi IrysChip–validated SVs supporting the results in this study are available from the corresponding author’s website [46]. Human reference genomes hg19 and GRCh38 are available from the Broad Institute [47] and UCSC [48], respectively. Snapshots of the GROM project code are available via the Open Science Framework [49] and the *GigaScience* database, *Giga*DB [50].

## Availability and requirements

Project name: GROM

Project home page: https://osf.io/6rtws/

Code DOI: 10.17605/OSF.IO/6RTWS

Operating system: Linux

Programming language: C

Other requirements: see manual in the distribution

License: GNU General Public License v2

## Additional files

Additional file 1: Supplementary tables. Benchmark results (Supplementary Tables S1–S6), run time comparisons (Supplementary Tables S7–S8), GROM-specific SVs overlapping genes (Supplementary Table S9), and duplicate read filtering comparison (Supplementary Table S10).

## Abbreviations

CNV: copy number variant; DGV-GS: Database of Genomic Variants–Gold Standard; GATK-HC: GATK HaplotypeCaller; GIAB: Genome In A Bottle; GROM: Genome Rearrangement OmniMapper; Mills-GS: Mills–Gold Standard; SNV: single nucleotide variant; SV: structural variant; WGS: whole genome sequencing.

## Competing interests

The authors declare that they have no competing financial interests.

## Author contributions

A.G. and S.D.S. conceived the project. S.D.S. designed and wrote the algorithm, with contributions from J.K.K. and A.G. S.D.S., J.K.K., and A.G. analyzed results. S.D.S. and A.G. wrote the manuscript with input from all authors. A.G. supervised the project and secured funding from startup and grant funds.

## Supplementary Material

GIGA-D-17-00105_Original-Submission.pdf

GIGA-D-17-00105_Revision-1.pdf

GIGA-D-17-00105_Revision-2.pdf

Response-to-Reviewer-Comments_Original-Submission.pdf

Response-to-Reviewer-Comments_Revision-1.pdf

Reviewer-1-Report-(Original-Submission).pdf

Reviewer-1-Report-(Revision-1).pdf

Reviewer-2-Report-(Original-Submission).pdf

Supplementary Tables
