# Supplementary material for: Lightning-fast genome variant detection with GROM
Source: Gigascience. 2017 Sep 18;6(10):1–7. doi: 10.1093/gigascience/gix091 (PMC5737730; doi:10.1093/gigascience/gix091)
Supplement: GIGA-D-17-00105_Revision-2.pdf [file gix091_giga-d-17-00105_revision-2.pdf]

GigaScience
Lightning-fast genome variant detection with GROM
--Manuscript Draft--

**Manuscript Number:**	GIGA-D-17-00105R2
**Full Title:**	Lightning-fast genome variant detection with GROM
**Article Type:**	Technical Note
**Funding Information:**	Directorate for Biological Sciences (1458202) — Dr. Andrey Grigoriev
**Abstract:**	Background: Current human whole genome sequencing projects produce massive amounts of data, often creating significant computational challenges. Different approaches have been developed for each type of genome variant and method of its detection, necessitating users to run multiple algorithms to find variants.<br><br>Results: We present GROM (Genome Rearrangement OmniMapper), a novel comprehensive variant detection algorithm accepting aligned files as input and finding SNVs, indels, structural variants (SVs), and copy number variants (CNVs). We show that GROM outperforms state-of-the-art methods on seven validated benchmarks using two whole genome sequencing (WGS) datasets. Additionally, GROM boasts lightning fast run times, analyzing a 50x WGS human dataset (NA12878) on commonly available computer hardware in 11 minutes, more than an order of magnitude (up to 72 times) faster than tools detecting a similar range of variants. GROM is also able to detect CNVs, SNVs and indels in non-paired read WGS libraries, as well as SNVs and indels in whole exome or RNA sequencing datasets.<br><br>Conclusion: Addressing the needs of big data analysis, GROM combines in one algorithm SNV, indel, SV, and CNV detection providing superior speed, sensitivity, and precision. GROM is also able to detect CNVs, SNVs and indels in non-paired read WGS libraries, as well as SNVs and indels in whole exome or RNA sequencing datasets.
**Corresponding Author:**	Andrey Grigoriev<br>Rutgers University<br>Camden, NJ UNITED STATES
**Corresponding Author Secondary Information:**	
**Corresponding Author's Institution:**	Rutgers University
**Corresponding Author's Secondary Institution:**	
**First Author:**	Sean D Smith
**First Author Secondary Information:**	
**Order of Authors:**	Sean D Smith<br>Joseph K Kawash<br>Andrey Grigoriev
**Order of Authors Secondary Information:**	
**Response to Reviewers:**	Reviewer's comment:<br><br>1. With respect to the comment 6 I gave in my previous feedback, the authors are correct that there exists no validated translocation dataset for either NA12878 or HX1, so it's acceptable if the authors skip benchmarking the translocations detected by GROM by stating in the main text the reason why they have skipped benchmarking translocation, but not other variant types. However, the real problem is, by not reporting the number of translocations detected by GROM in the paper, the authors failed to provide any evidence to support the claim in abstract that GROM is capable to detect

Powered by Editorial Manager® and ProduXion Manager® from Aries Systems Corporation

	translocation.<br><br>Response:<br>We have added the following sentences to the Results to address this:<br><br>"GROM also detected 545 and 472 translocation events in the NA12878 and HX1, respectively. However, these events were not included in the benchmarking due to the lack of validated translocation datasets for either genome.<br><br><br>We have also modified the Abstract to indicate GROM's applicability to the datasets other than WGS, as previously requested.
**Additional Information:**	
**Question**	**Response**
Are you submitting this manuscript to a special series or article collection?	No
**Experimental design and statistics**<br><br><br>Full details of the experimental design and statistical methods used should be given in the Methods section, as detailed in our Minimum Standards Reporting Checklist. Information essential to interpreting the data presented should be made available in the figure legends.<br><br><br>Have you included all the information requested in your manuscript?	Yes
**Resources**<br><br><br>A description of all resources used, including antibodies, cell lines, animals and software tools, with enough information to allow them to be uniquely identified, should be included in the Methods section. Authors are strongly encouraged to cite Research Resource Identifiers (RRIDs) for antibodies, model organisms and tools, where possible.<br><br><br>Have you included the information requested as detailed in our Minimum Standards Reporting Checklist?	Yes
**Availability of data and materials**<br><br><br>All datasets and code on which the conclusions of the paper rely must be either included in your submission or deposited in publicly available repositories (where available and ethically appropriate), referencing such data using a unique identifier in the references and in	Yes

the “Availability of Data and Materials” section of your manuscript.<br><br>Have you have met the above requirement as detailed in our Minimum Standards Reporting Checklist?	

Powered by Editorial Manager® and ProduXion Manager® from Aries Systems Corporation

Manuscript Click here to download Manuscript smith-etal-revised.docx

Lightning-fast genome variant detection with GROM

Sean D. Smith[1], Joseph K. Kawash[1], Andrey Grigoriev[1]

[1]Department of Biology, Center for Computational and Integrative Biology, Rutgers University, 315 Penn St, Camden 08102, NJ, USA

*To whom correspondence should be addressed: Tel. +856 225 2960. Fax +856 225 6312. Email: andrey.grigoriev@rutgers.edu

Email: ss1917@camden.rutgers.edu (Sean D. Smith), jokawash@scarletmail.rutgers.edu (Joseph K. Kawash), and andrey.grigoriev@rutgers.edu (Andrey Grigoriev).

Abstract

Background: Current human whole genome sequencing projects produce massive amounts of data, often creating significant computational challenges. Different approaches have been developed for each type of genome variant and method of its detection, necessitating users to run multiple algorithms to find variants.

Results: We present GROM (Genome Rearrangement OmniMapper), a novel comprehensive variant detection algorithm accepting aligned read files as input and finding SNVs, indels, structural variants (SVs), and copy number variants (CNVs). We show that GROM outperforms state-of-the-art methods on seven validated benchmarks using two whole genome sequencing (WGS) datasets. Additionally, GROM boasts lightning fast run times, analyzing a 50x WGS human dataset (NA12878) on commonly available computer hardware in 11 minutes, more than an order of magnitude (up to 72 times) faster than tools detecting a similar range of variants.

Conclusion: Addressing the needs of big data analysis, GROM combines in one algorithm SNV, indel, SV, and CNV detection providing superior speed, sensitivity, and precision. GROM is also able to detect CNVs, SNVs and indels in non-paired read WGS libraries, as well as SNVs and indels in whole exome or RNA sequencing datasets.

Keywords: variant detection, GROM, SNVs, structural variants, indels, copy number variants, whole genome sequencing

FINDINGS

Introduction

The 1,000 Genomes Project [1] was launched in 2008 with a goal of producing and analyzing whole genome sequencing (WGS) for 1,000 genomes. By 2016 decreasing costs and increasing sequencing throughput have led to an exponential increase in the size and scope of WGS projects from Human Longevity, Inc.'s 10,000 publicly available WGS genomes [2] to UK's 100,000 Genomes Project (https://www.genomicsengland.co.uk/) to even larger, though less-clearly defined, sequencing projects involving 1,000,000 participants proposed in the US (Precision Medicine Initiative, https://www.nih.gov/precision-medicine-initiative-cohort-program and Million Veteran Program, https://www.research.va.gov/mvp/) and China (http://www.genomics.cn). Such projects produce massive amounts of data, straining computational resources and requiring much faster methods than current capabilities [3].

Comprehensive analysis of genomic differences requires detection of a wide range of variants including single nucleotide variations (SNVs), indels (insertions and deletions <50 bases), and larger copy number variants (CNVs) and structural variants (SVs), which include deletions, duplications, insertions, inversions, and translocations. Methods have been developed for each type of variant, subsequently, a typical WGS analysis workflow requires running multiple algorithms. A recent pipeline, SpeedSeq [4], focused on reducing the computational resources needed for WGS analysis, though still employed four variant detection algorithms. This can be wasteful of computational resources due to repetitive input/output and analysis of the same read sequences by several algorithms.

We present our method, GROM (Genome Rearrangement Omni-Mapper), a novel comprehensive method of variant detection, combining mismatch, split-read, read pair, and read depth WGS evidence. GROM boasts lightning-speed runtimes, an order of magnitude faster than state-of-the-art variant detection pipelines. While drastically reducing computational time, GROM detects SNVs, indels, SVs, and CNVs in a single algorithm and provides superior overall variant detection compared to commonly employed algorithms.

Algorithm

Differences in variant types (Figure 1) have resulted in separate algorithms designed for a limited range of variants. GROM achieves fast, comprehensive variant analysis via a compact workflow (Figure 2) efficiently analyzing and gathering information at each reference base in one pass through a BAM file. Base information includes average mapping and base qualities; overlapping discordant pairs, unmapped mate reads, and split-reads; and read depth. Discordant pairs are identified based on abnormal read orientation or abnormal insert size. GROM determines abnormal insert size based on a sample of 10 million paired reads. Since insert size distributions tend to have right skewness, GROM calculates the median insert size and uses a rank-based method to determine abnormal insert size thresholds corresponding to 3 standard deviations from the median under a normal distribution (after outliers more than 5x the median insert size have been filtered). Each read with a split mapping, indel, discordant mate, or unmapped mate contributes breakpoint evidence to each potential reference base breakpoint. For simple cases such as a 2-base deletion within a read, there is one potential reference base start breakpoint and one potential reference base end breakpoint. Other cases may have less precise breakpoints, such as a read from a discordant deletion pair (abnormally large insert size). In this case, the exact

breakpoint is unknown and a potential breakpoint is recorded for each reference base consistent with forming a concordant pair in the sample, where a concordant pair corresponds to insert sizes $\geq i_{min}$ and $\leq i_{max}$, where i_{min} and i_{max} represent the minimum and maximum insert size thresholds, respectively (Figure 3). Using the deletion example in Figure 3, a breakpoint distant from both reads would necessitate an insert size that is too large to be consistent with a concordant pair (and the source DNA fragment), and thus would not be a potential breakpoint. When soft-clipping (≥5 bases) or a split-read (each mapped split ≥20 bases) occurs in the potential breakpoint region, the reference base immediately adjacent to the soft-clipping or split-read is recorded as a potential breakpoint and other potential breakpoints are recorded with half-weighting. This enables base resolution of breakpoints while limiting a single aberrant read mapping from misidentifying the true breakpoint.

Base by base of the reference, breakpoint evidence is stored for each distinct indel or SV. In some cases, it is difficult to distinguish variants. For instance, two heterozygous deletions may overlap and have similar start and end breakpoints and similar lengths. Thus, for each potential breakpoint we cluster read evidence by variant type and length. Such clustering can be a computationally intensive task. We use the following efficient method.

We define a cluster or breakpoint cluster as a specific reference base location with a set of reads supporting a breakpoint at that location for a specific indel or SV type (deletion, duplication, etc.) of a certain length. A read from a discordant pair provides imprecise breakpoints and thus may be a member of multiple clusters, one cluster per reference location. A read is placed into an

existing breakpoint cluster if the read and cluster support the same indel or SV type and the variant lengths are close, i.e.,

$$|L_{bc} - L_{disc}| \leq (i_{max} - i_{min} + i_{median} - 2L_r)\left(1 + \frac{1}{x_{bc}}\right) \quad (1),$$

where L_{bc} is the mean indel or SV length for the breakpoint cluster, L_{disc} is the length of the indel or SV pertaining to the candidate read, L_r is the read length, x_{bc} is the number of previously recorded reads supporting the breakpoint cluster, and i_{max}, i_{min}, and i_{median} are the maximum, minimum, and median concordant pair lengths, respectively. If a candidate read does not fit in any existing breakpoint clusters, a new cluster is created. If a candidate read fits in more than one breakpoint cluster at the same reference position, the breakpoint cluster with the most reads is chosen. This method is efficient and has the benefit of a read being considered in multiple clusters.

Additionally, the number of previously recorded reads influences whether a read is added to a breakpoint cluster because we expect our estimated (averaged) variant length to be closer to the true SV length as supporting reads are incorporated into the SV length average. For example, in Eq. 1 let insert size statistics be such that $i_{max} - i_{min} + i_{median} - 2L_r = 500$, let an SV be a deletion of 1200 bases and let our first discordant pair indicate an SV of length $L_{disc} = 1700$. One read is a poor estimate of the true SV length. Thus, in our example, the second read's SV length may differ from the first read's SV length by 1000 bases, $|1700 - L_{disc}| \leq 1000$. However, as the number of supporting reads increase, we expect the average SV length (L_{bc}) to converge to the true SV length of 1200, at which point we will not add the read as evidence unless its estimated

SV length (L_{disc}) is within 500 bases of the true SV length, $|1200 - L_{disc}| \leq 500*(1 + \varepsilon)$, where $\varepsilon << 1$.

For each reference base, a mismapping probability, p_{bc}, is calculated for each possible SNV, indel, and SV. p_{bc} is the binomial probability of at least x_{bc} reads supporting the breakpoint cluster given n_{bc} read depth and a mapping quality threshold m. Thus, p_{bc} indicates the likelihood that all of the supporting reads are mismappings. Read depth includes all mapped reads, unsequenced segments between concordant pairs, and potential breakpoints, and thus is an estimate of physical coverage. Physical coverage provides a more comprehensive representation of genome coverage than read coverage. It also helps GROM define deletion and duplication breakpoints when soft-clipping is unavailable as a decrease in coverage will affect breakpoint probability estimates. The mapping quality threshold m indicates the probability of a read mismapping, $p = 10^{-m/10}$. Thus, p_{bc} is given as:

$$p_{bc} = \Pr(\mathrm{X} \geq x) = 1 - \sum_{k=0}^{x-1} \binom{n}{k} p^k q^{n-k} \qquad (2),$$

where $q = 1 - p$. To reduce computational time, binomial probability tables are precomputed and stored as data files. GROM will compute additional probability data files if the default mapping quality threshold ($m = 20$) is adjusted.

Potential indel and SV breakpoints are retained for further analysis. After processing reads for a chromosome (or the whole genome for translocations), GROM identifies indels and SVs with matching start and end breakpoints. Matching SV breakpoints must meet the following criteria

$$|B_S + L_S - B_e| \leq \ c \times (i_{max} - i_{min}) \quad (3),$$

$$|B_e - L_e - B_s| \leq c \times (i_{max} - i_{min}) \quad (4),$$

where c = 3/8, B_s and B_e are the start and end breakpoints, respectively, and L_s and L_e are the average variant length of reads supporting the start or end breakpoints, respectively. Matching translocation breakpoints follow the same concept modified due to the start and end breakpoints occurring on different chromosomes,

$$|M_S - B_e| \leq \ c \times (i_{max} - i_{min}) \quad (5),$$

$$|M_e - B_s| \leq c \times (i_{max} - i_{min}) \quad (6),$$

where c = 3/8, B_s and B_e are the start and end breakpoints, respectively, and M_s and M_e are the average mate read reference of reads supporting the start or end breakpoints, respectively.

Mixed libraries/BAM files, e.g., with insert size distributions appreciably different as to affect the Eqs. 3-6 for matching breakpoints, or libraries containing paired-end with mate-pair data, require separate runs of GROM. Also, GROM can analyze exome or RNA sequencing reads with detection limited to SNVs and indels.

GROM will also work for libraries of non-paired reads using (in addition to finding SNVs and SVs within reads) our earlier method for finding copy number variants (CNVs), GROM-RD [5]. GROM-RD also performs well compared to the standard tools such as CNVnator [6]. GROM and GROM-RD have the same foundation of collecting information for each reference base but GROM-RD detects CNVs based on read depth, where low or high coverage is evidence of a

deletion or duplication, respectively. This method is complementary to the core GROM approach described above.

GROM is able to simultaneously perform duplicate filtering, its duplicate filter is conceptually similar to Picard's MarkDuplicates [7] and SAMtools rmdup [8], which have been shown to have similar performance. Duplicate filtering may improve predictive accuracy relative to no filtering [9]. GROM provides an option to include such filtering, if necessary. GROM filters read pairs with identical orientation and external mapping coordinates, retaining the pair with highest mapping quality. Unlike SAMtools, GROM and Picard's MarkDuplicates are able to filter duplicates with reads mapping to different chromosomes and adjust external coordinates based on soft-clipping [9]. For the sake of speed optimization and one-pass analysis, soft-clipping is not considered for a read's mate.

Results

We compared GROM's performance to four commonly used algorithms, GATK HaplotypeCaller (GATK-HC) [10], SAMtools [8], LUMPY [11], and Manta [12] using two extensively validated human WGS datasets, 51x NA12878 "platinum" genome [13] and 68x HX1, a recent Chinese genome [14]. GATK-HC, considered a gold standard in SNV/indel detection, has been shown to outperform state-of-the-art algorithms [15], and SAMtools is present in most pipelines. Because GROM integrates multiple lines of evidence, we also specifically compared it with a similar SV tool in the SpeedSeq pipeline (SpeedSeq, RRID:SCR_000469), LUMPY, shown to outperform other algorithms [11], such as DELLY (DELLY, RRID:SCR_004603)[16], Pindel (Pindel, RRID:SCR_000560)[17], and GASVPro

(GASVPro, RRID:SCR_005259)[18]. As part of a 10,000 genome sequencing study, presently the largest human WGS variant study, a comparison of seven SV detection algorithms (BreakDancer [19], DELLY [16], GenomeSTRiP [20], LUMPY [11], Manta [12], MatchClip2 [21], and Pindel [17]), showed Manta performed the best for SV detection [2]. We evaluated SNV and indel detection with the Illumina Platinum pedigree-validated benchmark sets [13]. GROM exhibited the highest SNV and insertion indel sensitivity and precision and highest deletion indel sensitivity when compared to GATK-HC and SAMtools for the NA12878 genome (Supplementary Table 1). SVs are notoriously difficult to reliably detect [2]. Thus, we extensively analyzed GROM's performance using four benchmark sets for NA12878: Database of Genomic Variants Gold Standard (DGV-GS, deletions and duplications) [22], Mills Gold Standard (Mills-GS; deletions, duplications, and insertions) [23], Genome in a Bottle (GIAB, deletions and insertions) [24]; and Pendleton PacBio (deletions and inversions) [25]. And, we utilized three deletion and duplication benchmark sets for HX1: DGV-GS, Shi PacBio [14], and Shi IrysChip [14] (see Methods section for a more complete description of benchmark/validation sets). A summary of the deletion and duplication comparison with LUMPY and Manta, indicated superior deletion and duplication detection (Supplementary Table 2) with GROM highest in 10 of 14 deletion (Supplementary Table 3) and 7 of 10 duplication (Supplemental Table 4) metrics (sensitivity and precision) across the benchmark datasets. Additionally, GROM was highest in all inversion (Supplemental Table 5) and insertion (Supplemental Table 6) metrics. GROM also detected 545 and 472 translocation events in the NA12878 and HX1, respectively. However, these events were not included in the benchmarking due to the lack of validated translocation datasets for either genome.

With dropping sequencing costs and growing data throughput, it is imperative to reduce the computational costs of big data analysis. GROM was 1.7x (NA12878) and 2.1x (HX1) faster than the next fastest algorithm, Manta (Supplementary Table 7). Since typical analyses involve running separate algorithms for SNV/indel and SV detection, we compared a simple 24-thread parallelized GROM version (allocating a thread per 1/24 of the genome) with the fastest and best-performing two-algorithm workflow (GATK-HC/Manta). Strikingly, GROM ranged from 24x (HX1, no duplicate filtering) to 72x (NA12878 with duplicate filtering) faster than a combination of 22-thread GATK-HC/2-thread Manta (Supplementary Table 8), drastically reducing variant detection and duplicate filtering from 41% to <1% of a typical WGS analysis pipeline (Figure 4). For 1,000 genomes on a 24-thread server, it may literally save years of computation.

Comparing the variants predicted by different tools, we identified 33 validated NA12878 SVs detected by GROM (but unreported by LUMPY and Manta) that overlapped genes, and ranked them using the number of independent validations (Supplementary Table 9). A variant was considered validated if it occurred in at least one of the NA12878 benchmarks corresponding to the SV type (DGV-GS, Mills-GS, GIAB, Pendleton PacBio for deletions; DGV-SV, Mills-GS for duplications; Mills-GS, GIAB for insertions; and Pendleton PacBio for inversions).

Among these variants, we noted four deletions with significant health-related impact for NA12878: RHD, GSTM1, IFI16, and UGT2B17 (Figure 5). GROM predicted a deletion spanning the entire RHD gene, one of two genes responsible for Rh blood group antigens [26]. Decreased copy numbers or null genotype of GSTM1 have been associated with hepatotoxicity

[27] and higher risk of many cancers including lung cancer [28], gastric cancer [29], and bladder cancer [30]. UGT2B17 copy number variation has been associated with changes in bone mineral density and in risk of osteoporosis [31]. IFI16 is involved in viral defense [32] and p53-mediated apoptosis [33, 34].

Additionally, GROM provides an option to include duplicate filtering. This leads to minor accuracy gains in a number of cases (see example in Supplementary Table 10) and achieves additional speedup (Supplementary Table 8). Lastly, we have summarized GROM's relative performance in Table 1.

Table 1 Comparison of GROM and leading algorithms'variant detection accuracy and run time. Performance based on sensitivity and precision rankings (1=highest, 3=lowest) averaged across benchmarks for NA12878 and HX1. Bold text indicates the best performing algorithm in each category. A dash sign indicates that an algorithm does not detect variant type.

Algorithm		GATK-HC	SAMtools	LUMPY	Manta	GROM
SNV		2	3	-	-	**1**
Indel	Deletion	**1**	3	-	-	**1**
	Insertion	2	3	-	-	**1**
SV	Deletion	-	-	2	3	**1**
	Duplication	-	-	2	2	**1**
	Insertion	-	-	-	2	**1**
	Inversion	-	-	3	2	**1**
Run Time		4	5	3	2	**1**

METHODS

All timings were performed on an Intel Xeon E5-2690 v3 processor, 2.60 GHz, with 24 threads and 128 GB RAM.

Rankings in Table 1 and Supplementary Table 2 were based on average ranking across benchmarks (1-highest to 3-lowest). Ranking for each benchmark was based on sensitivity and precision values in Supplementary Tables 3-6. For instance, GROM had the highest value for 10, second highest for two, and lowest for two of the 14 deletion sensitivity and precision benchmarks (average benchmark rank 1.4) Subsequently, the algorithms were ranked after sorting by their average benchmark ranking, resulting in deletion rankings of GROM, 1; LUMPY, 2; and Manta, 3 (as shown in Table 1).

Unlike most SV variant callers, GROM is able to analyze datasets with single or paired reads. However, all SV tests included only paired reads since most of the other callers operate on those.

While state-of-the-art detection methods for SNVs and indels have been deemed adequate for the clinical setting, SV detection is notably more difficult [2]. Additionally, synthetic datasets have suffered from oversimplifications and misleading conclusions [2]. Thus, we extensively analyzed GROM's SV detection performance using four validation benchmark sets for NA12878:

1) Database of Genomic Variants Gold Standard (deletions and duplications) [22] in Supplementary Tables 2-4;

2) Mills Gold Standard (deletions, duplications, and insertions) [23] in Supplementary Tables 2-5;

3) Genome in a Bottle (deletions and insertions) [24] in Supplementary Tables 2,3,5; and

4) Pendleton PacBio (deletions and inversions) [25] in Supplementary Tables 2,3,6.

Additionally, we utilized three deletion and duplication benchmark sets for HX1: DGV-GS (as above), Shi PacBio, and Shi IrysChip [14] in Supplementary Tables 2-4. For NA12878 DGV-GS benchmarks, all deletions and duplications with the “NA12878” tag were extracted from the DGV-GS. The HX1 DGV-GS benchmarks were created by extracting deletions and duplications with the “Asian” tag. To obtain a benchmark set of common Asian variants, deletions and duplications with less than 200 “Asian”-tagged samples were filtered.

To limit potential biases, we selected benchmarks covering a range of technologies, including Illumina, PacBio, and IrysChip, and inclusive of multiple variant detection algorithms (Illumina platinum pedigree-validated, DGV-GS, Mills-GS, and GIAB). Indels were defined as deletions and insertions <50 bases, whereas SVs were ≥50 bases. To identify true positives, indel benchmarking required variant call breakpoints within 2 bases of the benchmark. Insertion SV calls within 10 bases of the benchmark were considered true. All other SV benchmarking required 50% (10% for IrysChip due to low resolution) reciprocal overlap of a variant call and the benchmark. Some false positives may potentially be true positives not represented in the benchmark. To limit false positives due to unrepresented calls, for each SV type (excluding insertions where the length is often unknown) we ignored SV calls smaller or larger than a particular benchmark’s shortest or longest SV, respectively.

NA12878 and HX1 Illumina platinum fasta files were mapped to human references hg19 and GRCh38, respectively, using BWA mem [35], version 0.7.15, with the -M parameter to mark shorter read splits as secondary. Duplicate filtering comparisons were performed using default parameters for SAMtools [8], version 1.3.1 and Sambamba [36], version 0.6.4. GATK version

3.6.0 HaplotypeCaller (GATK , RRID:SCR_001876) [10], SAMtools (SAMTOOLS , RRID:SCR_002105) [8], LUMPY (LUMPY, RRID:SCR_003253)[11] (version 0.2.11), and Manta [12] (version 1.0.1) were run with default parameters.

Conclusion

Our extensive performance analysis indicates GROM achieves superior variant detection and is significantly faster than current state-of-the-art methods by incorporating comprehensive variant detection (SNV, indel, SV, CNV), duplicate filtering, and multithreading in one algorithm. GROM's superior variant detection makes it valuable for WGS analysis projects of all sizes, and its "lightning" fast speed is especially critical for keeping pace with increasingly higher sequencing throughput and larger data projects.

Availability and requirements

Project name: GROM

Project home page: https://osf.io/6rtws/

Code DOI: 10.17605/OSF.IO/6RTWS

Operating system: Linux

Programming language: C

Other requirements: See manual in the distribution

License: GNU General Public License v2

Additional files

Additional file 1: Supplementary tables. Benchmark results (Supplementary tables 1-6), run time comparisons (Supplementary tables 7-8), GROM-specific SVs overlapping genes (Supplementary table 9), and duplicate read filtering comparison (Supplementary table 10). (DOCX)

Abbreviations

CNV, copy number variant; DGV-GS, Database of Genomic Variants – Gold Standard; GATK-HC, GATK HaplotypeCaller; GIAB, Genome In A Bottle; GROM, Genome Rearrangement OmniMapper; Mills-GS, Mills – Gold Standard; SNV, single nucleotide variant; SV, structural variant; WGS, Whole genome sequencing.

Acknowledgements

The authors thank Kevin Abbey, Hui-Jou Chou, Spyros Karaiskos, Ian Biluck (all from Rutgers), for excellent technical help throughout the development and testing process. The authors thank Tal Nevo, Benzi Galili, and Erez Valen (all from ScaleMP, Inc.) for discussions, suggestions and code contributions used in the parallel version of GROM. This work was supported by the National Science Foundation (award DBI-1458202 to A.G.).

Availability of data and materials

NA12878 raw short-read Illumina platinum WGS data, as well as pedigree-validated SNVs and indels, supporting the results in this study are available from the Database of Genotypes and Phenotypes under accession number phs001224.v1.p1 [37]. HX1 raw short-read Illumina WGS data supporting the results in this study are available from the National Center for Biotechnology

Information (NCBI) Sequence Read Archive (SRA), study PRJNA301527 [38]. DGV-GS validated SVs supporting the results in this study are available from the Database of Genomic Variants website [39]. Mills-GS validated SVs supporting the results in this study are available as Supplementary Table 5 in the associated paper [23]. GIAB validation data supporting the results in this study are available from NCBI at separate locations for deletions (ver. 3.3.1) [40] and insertions [41]. Pendleton PacBio validated deletions and inversions supporting the results in this study are available as Supplementary Tables 5 and 6, respectively in the associated paper [25]. Shi PacBio and Shi IrysChip validated SVs supporting the results in this study are available from the corresponding authors website [42]. Human reference genomes hg19 and GRCh38 are available from the Broad Institute [43] and UCSC [44], respectively. Snapshots of the GROM project code are available via the OSF [45] and the *GigaScience* GigaDB database[46].

Authors contributions

A.G. and S.D.S conceived the project. S.D.S designed and wrote the algorithm, with contributions from J.K.K. and A.G. S.D.S, J.K.K. and A.G. analyzed results. S.D.S and A.G. wrote the manuscript with input from all authors. A.G. supervised the project and secured funding from startup and grant funds.

Competing interests

The authors declare that they have no competing financial interests.

References

1. Genomes Project C, Abecasis GR, Altshuler D, Auton A, Brooks LD, Durbin RM, et al. A map of human genome variation from population-scale sequencing. Nature. 2010;467 7319:1061-73. doi:10.1038/nature09534.
2. Telenti A, Pierce LC, Biggs WH, di Iulio J, Wong EH, Fabani MM, et al. Deep sequencing of 10,000 human genomes. Proc Natl Acad Sci U S A. 2016;113 42:11901-6. doi:10.1073/pnas.1613365113.
3. Stephens ZD, Lee SY, Faghri F, Campbell RH, Zhai C, Efron MJ, et al. Big data: astronomical or genomical? PLoS biology. 2015;13 7:e1002195.
4. Chiang C, Layer RM, Faust GG, Lindberg MR, Rose DB, Garrison EP, et al. SpeedSeq: ultra-fast personal genome analysis and interpretation. Nature Methods. 2015;12 10:966-8. doi:10.1038/Nmeth.3505.
5. Smith SD, Kawash JK and Grigoriev A. GROM-RD: resolving genomic biases to improve read depth detection of copy number variants. PeerJ. 2015;3:e836. doi:10.7717/peerj.836.
6. Abyzov A, Urban AE, Snyder M and Gerstein M. CNVnator: an approach to discover, genotype, and characterize typical and atypical CNVs from family and population genome sequencing. Genome Res. 2011;21 6:974-84. doi:10.1101/gr.114876.110.
7. Institute B. Picard Tools. 2.5 ed. 2016.
8. Li H, Handsaker B, Wysoker A, Fennell T, Ruan J, Homer N, et al. The Sequence Alignment/Map format and SAMtools. Bioinformatics. 2009;25 16:2078-9. doi:10.1093/bioinformatics/btp352.
9. Ebbert MT, Wadsworth ME, Staley LA, Hoyt KL, Pickett B, Miller J, et al. Evaluating the necessity of PCR duplicate removal from next-generation sequencing data and a comparison of approaches. BMC Bioinformatics. 2016;17 Suppl 7:239. doi:10.1186/s12859-016-1097-3.
10. DePristo MA, Banks E, Poplin R, Garimella KV, Maguire JR, Hartl C, et al. A framework for variation discovery and genotyping using next-generation DNA sequencing data. Nature Genetics. 2011;43 5:491-+. doi:10.1038/ng.806.
11. Layer RM, Chiang C, Quinlan AR and Hall IM. LUMPY: a probabilistic framework for structural variant discovery. Genome Biology. 2014;15 6: R84. doi: 10.1186/gb-2014-15-6-r84.
12. Chen XY, Schulz-Trieglaff O, Shaw R, Barnes B, Schlesinger F, Kallberg M, et al. Manta: rapid detection of structural variants and indels for germline and cancer sequencing applications. Bioinformatics. 2016;32 8:1220-2. doi:10.1093/bioinformatics/btv710.
13. Eberle MA, Fritzilas E, Krusche P, Kallberg M, Moore BL, Bekritsky MA, et al. A reference data set of 5.4 million phased human variants validated by genetic inheritance from sequencing a three-generation 17-member pedigree. Genome Res. 2017;27 1:157-64. doi:10.1101/gr.210500.116.
14. Shi LL, Guo YF, Dong CL, Huddleston J, Yang H, Han XL, et al. Long-read sequencing and de novo assembly of a Chinese genome. Nature Communications. 2016;7:12065. doi: 10.1038/ncomms12065.
15. Yi M, Zhao YM, Jia L, He M, Kebebew E and Stephens RM. Performance comparison of SNP detection tools with illumina exome sequencing data-an assessment using both family pedigree information and sample-matched SNP array data. Nucleic Acids Research. 2014 42(12):e101. doi: 10.1093/nar/gku392.
16. Rausch T, Zichner T, Schlattl A, Stutz AM, Benes V and Korbel JO. DELLY: structural variant discovery by integrated paired-end and split-read analysis. Bioinformatics. 2012;28 18:I333-I9. doi:10.1093/bioinformatics/bts378.
17. Ye K, Schulz MH, Long Q, Apweiler R and Ning Z. Pindel: a pattern growth approach to detect break points of large deletions and medium sized insertions from paired-end short reads. Bioinformatics. 2009;25 21:2865-71. doi:10.1093/bioinformatics/btp394.

18. Sindi SS, Onal S, Peng LKC, Wu HT and Raphael BJ. An integrative probabilistic model for identification of structural variation in sequencing data. Genome Biology. 2012;13 3: R22 doi:10.1186/Gb-2012-13-3-R22.

19. Chen K, Wallis JW, McLellan MD, Larson DE, Kalicki JM, Pohl CS, et al. BreakDancer: an algorithm for high-resolution mapping of genomic structural variation. Nat Methods. 2009;6 9:677-81. doi:10.1038/nmeth.1363.

20. Handsaker RE, Van Doren V, Berman JR, Genovese G, Kashin S, Boettger LM, et al. Large multiallelic copy number variations in humans. Nat Genet. 2015;47 3:296-303. doi:10.1038/ng.3200.

21. Wu Y, Tian L, Pirastu M, Stambolian D and Li H. MATCHCLIP: locate precise breakpoints for copy number variation using CIGAR string by matching soft clipped reads. Frontiers in Genetics. 2013;4 157 doi:10.3389/fgene.2013.00157.

22. MacDonald JR, Ziman R, Yuen RK, Feuk L and Scherer SW. The Database of Genomic Variants: a curated collection of structural variation in the human genome. Nucleic Acids Res. 2014;42 Database issue:D986-92. doi:10.1093/nar/gkt958.

23. Mills RE, Walter K, Stewart C, Handsaker RE, Chen K, Alkan C, et al. Mapping copy number variation by population-scale genome sequencing. Nature. 2011;470 7332:59-65. doi:10.1038/nature09708.

24. Zook JM, Chapman B, Wang J, Mittelman D, Hofmann O, Hide W, et al. Integrating human sequence data sets provides a resource of benchmark SNP and indel genotype calls. Nature Biotechnology. 2014;32 3:246-51. doi:10.1038/nbt.2835.

25. Pendleton M, Sebra R, Pang AW, Ummat A, Franzen O, Rausch T, et al. Assembly and diploid architecture of an individual human genome via single-molecule technologies. Nat Methods. 2015;12 8:780-6. doi:10.1038/nmeth.3454.

26. Wagner FF and Flegel WA. RHD gene deletion occurred in the Rhesus box. Blood. 2000;95 12:3662-8.

27. Singh HO, Lata S, Angadi M, Bapat S, Pawar J, Nema V, et al. Impact of GSTM1, GSTT1 and GSTP1 gene polymorphism and risk of ARV-associated hepatotoxicity in HIV-infected individuals and its modulation. Pharmacogenomics J. 2017;17 1:53-60. doi:10.1038/tpj.2015.88.

28. Yang H, Yang S, Liu J, Shao F, Wang H and Wang Y. The association of GSTM1 deletion polymorphism with lung cancer risk in Chinese population: evidence from an updated meta-analysis. Scientific reports. 2015;5:9392. doi:10.1038/srep09392.

29. Lao X, Peng Q, Lu Y, Li S, Qin X, Chen Z, et al. Glutathione S-transferase gene GSTM1, gene-gene interaction, and gastric cancer susceptibility: evidence from an updated meta-analysis. Cancer cell international. 2014;14 1:127. doi:10.1186/s12935-014-0127-3.

30. Norskov MS, Frikke-Schmidt R, Bojesen SE, Nordestgaard BG, Loft S and Tybjrg-Hansen A. Copy number variation in glutathione-S-transferase T1 and M1 predicts incidence and 5-year survival from prostate and bladder cancer, and incidence of corpus uteri cancer in the general population. Pharmacogenomics Journal. 2011;11 4:292-9. doi:10.1038/tpj.2010.38.

31. Yang TL, Chen XD, Guo Y, Lei SF, Wang JT, Zhou Q, et al. Genome-wide Copy-Number-Variation Study Identified a Susceptibility Gene, UGT2B17, for Osteoporosis. American Journal of Human Genetics. 2008;83 6:663-74. doi:10.1016/j.ajhg.2008.10.006.

32. Orzalli MH, Conwell SE, Berrios C, DeCaprio JA and Knipe DM. Nuclear interferon-inducible protein 16 promotes silencing of herpesviral and transfected DNA. Proceedings of the National Academy of Sciences of the United States of America. 2013;110 47:E4492-E501. doi:10.1073/pnas.1316194110.

33. Aglipay JA, Lee SW, Okada S, Fujiuchi N, Ohtsuka T, Kwak JC, et al. A member of the Pyrin family, IFI16, is a novel BRCA1-associated protein involved in the p53-mediated apoptosis pathway. Oncogene. 2003;22 55:8931-8. doi:10.1038/sj.onc.1207057.

34. Johnstone RW, Wei W, Greenway A and Trapani JA. Functional interaction between p53 and the interferon-inducible nucleoprotein IFI 16. Oncogene. 2000;19 52:6033-42. doi:DOI 10.1038/sj.onc.1204005.
35. Li H and Durbin R. Fast and accurate short read alignment with Burrows-Wheeler transform. Bioinformatics. 2009;25 14:1754-60. doi:10.1093/bioinformatics/btp324.
36. Tarasov A, Vilella AJ, Cuppen E, Nijman IJ and Prins P. Sambamba: fast processing of NGS alignment formats. Bioinformatics. 2015;31 12:2032-4. doi:10.1093/bioinformatics/btv098.
37. database of Genotypes and Phenotypes. https://www.ncbi.nlm.nih.gov/gap. Accessed April 24 2017.
38. NCBI Sequence Read Archive. https://www.ncbi.nlm.nih.gov/sra/. Accessed April 24 2017.
39. Database of Genomic Variants. dgv.tcag.ca.
40. Zook JM, Catoe D, McDaniel J, Vang L, Spies N, Sidow A, et al. Extensive sequencing of seven human genomes to characterize benchmark reference materials. Scientific data. 2016;3.
41. Parikh H, Mohiyuddin M, Lam HY, Iyer H, Chen D, Pratt M, et al. svclassify: a method to establish benchmark structural variant calls. BMC genomics. 2016;17 1:64.
42. Shi PacBio and Shi IrysChip validated SVs. http://hx1.wglab.org/data/cnv_sv/. Accessed April 24 2017.
43. hg19 Human Reference Genome, Broad Institute. https://software.broadinstitute.org/gatk/download/bundle. Accessed April 24 2017.
44. GRCh38 Human Reference Genome, UCSC. https://genome.ucsc.edu. Accessed April 24 2017.
45. Smith, Sean, and Andrey Grigoriev. 2017. “GROM.” Open Science Framework. July 28. http://doi.org/10.17605/OSF.IO/6RTWS
46. Smith, S, D; Kawash, J, K; Grigoriev, A (2017): Supporting data for "Lightning-fast genome variant detection with GROM" GigaScience Database. http://dx.doi.org/10.5524/100352

Figure1

Click here to download Figure GigaScience_smith_etal_figure1.docx

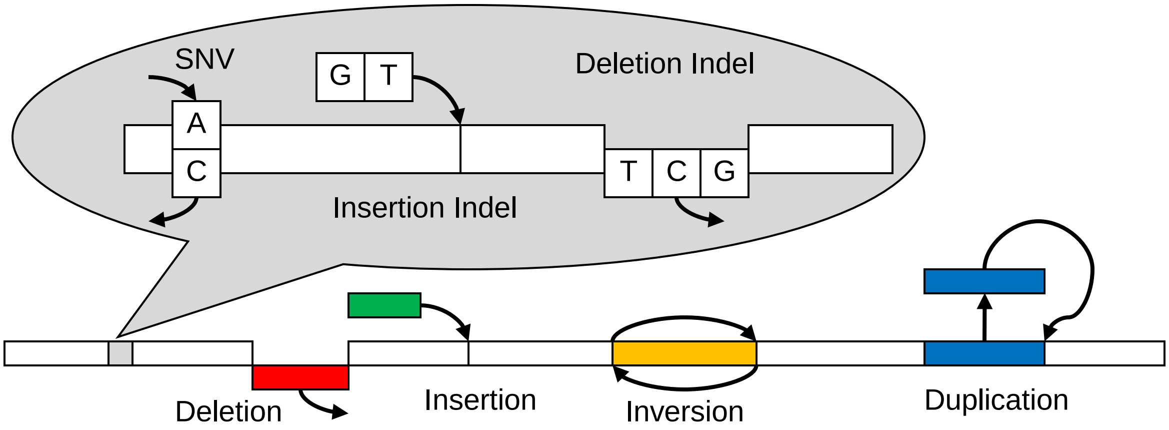

Figure 1. Examples of variants detected by GROM. GROM detects a comprehensive range of variants (SNVs, indels, deletions, insertions, inversions, and duplications). GROM also detects translocations spanning more than one chromosome (not shown).

Click here to download Figure GigaScience_smith_etal_figure2.docx

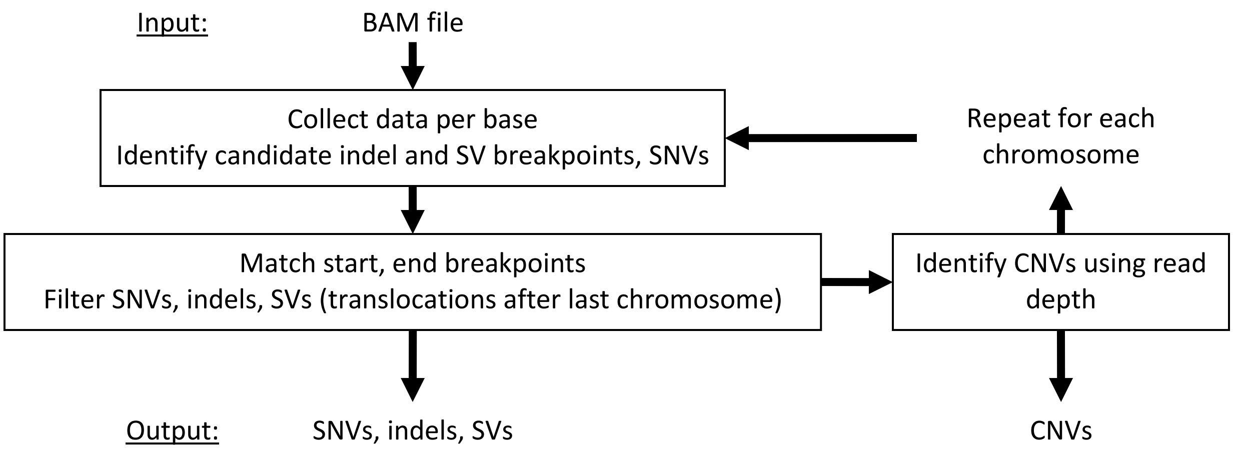

Figure 2 GROM workflow. GROM simultaneously collects data for each reference base and identifies candidate breakpoints and SNVs in one pass through a BAM file. After each chromosome, SNVs are filtered; start and end breakpoints are matched and filtered for each indel and SV type (excluding translocations); and CNVs are identified (using read depth).

Figure3 Click here to download Figure GigaScience_smith_etal_figure3.docx

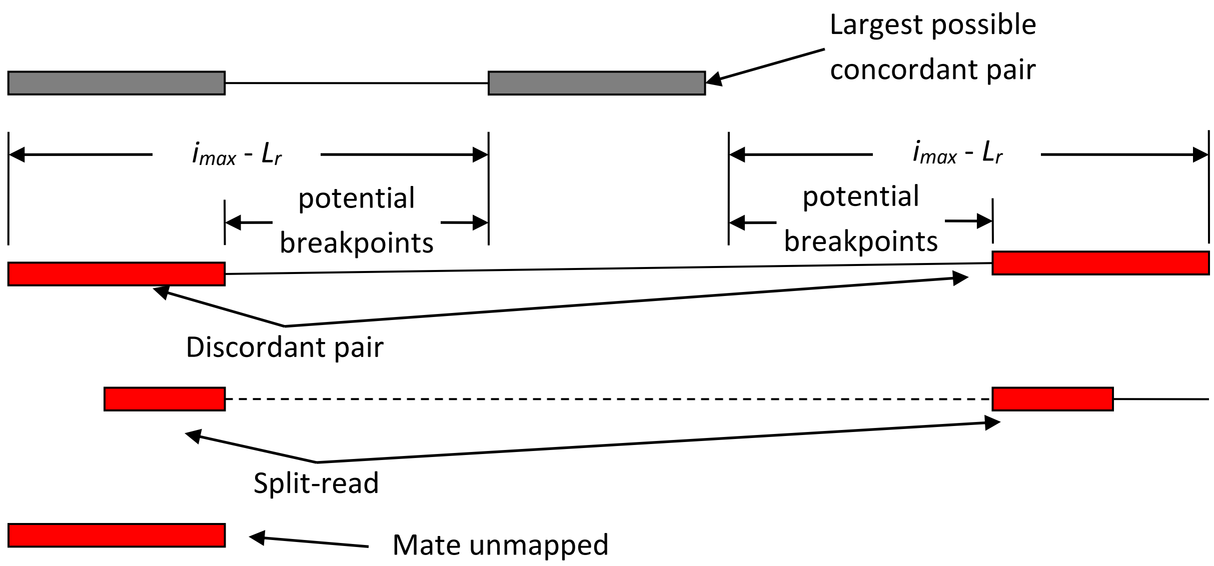

Figure 3 Example of SV evidence and potential breakpoints. GROM considers multiple input features at each reference base position to statstically determine the likelihood of a SNV, indel, SV, or CNV. Inputs in this example (discordant pairs, split-reads, and mate unmapped reads) are primarily used for SV detection. Discordant deletion pairs identified by insert size exceeding i_{max}. For discordant pairs, potential start and end breakpoints are recorded for each reference base capable of forming a concordant pair in the sample. L_r indicates read length.

Figure4 Click here to download Figure GigaScience_smith_etal_figure4.docx

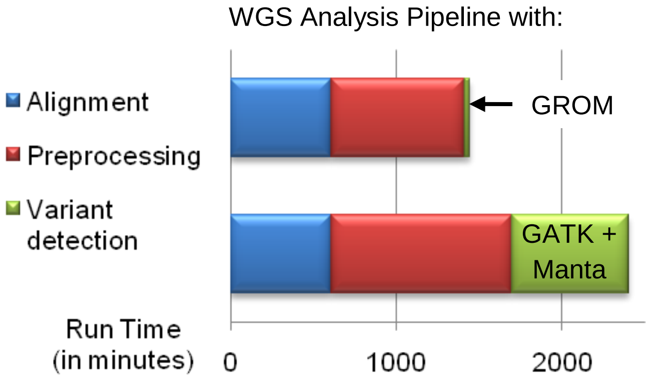

Figure 4. Total WGS pipeline timing on NA12878. GROM reduces WGS analysis time by drastically cutting run time for variant detection (green). It enables further speedup in preprocessing (red) by simultaneously performing an optional step, duplicate filtering. For visibility in the bar chart, GROM's variant detection run time was artificially increased 3-fold.

Click here to download Figure GigaScience_smith_etal_figure5.docx

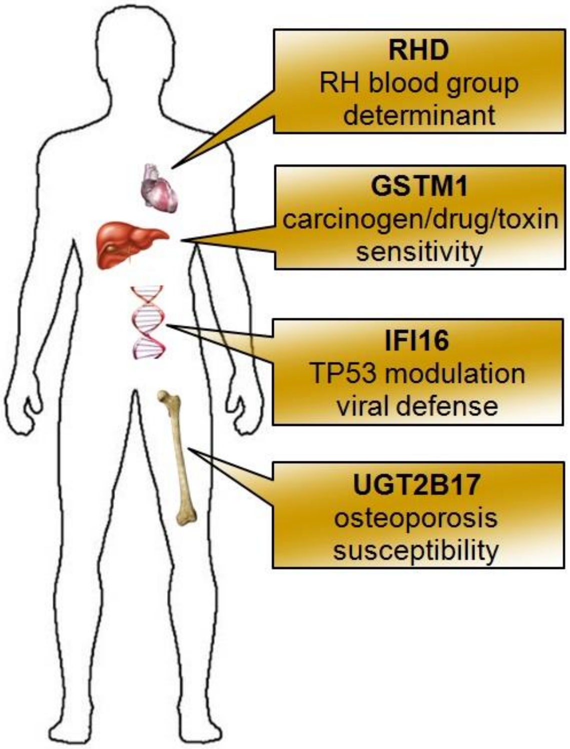

Figure 1 Example of genes overlapped by validated GROM-specific SVs. In example are four of 33 genes overlapped by validated SVs that were identified by GROM and unreported by LUMPY and Manta. Biological significance listed below gene.

Supplementary Tables

Click here to access/download
Supplementary Material
GigaScience_smith_etal_supp_tables.docx

 Click here to download Personal Cover cover-7sep.docx

Sep 7, 2017

Dear Editor,

We have revised the manuscript by addressing the remaining reviewers' concerns and by including the previously outlined change to the abstract.

We greatly appreciate you expediting the review process and hope you find the paper acceptable for publication in GigaScience.

On behalf of all authors,

Andrey Grigoriev
