# Supplementary material for: Lightning-fast genome variant detection with GROM
Source: Gigascience. 2017 Sep 18;6(10):1–7. doi: 10.1093/gigascience/gix091 (PMC5737730; doi:10.1093/gigascience/gix091)
Supplement: Supplementary Tables [file gix091_supp.docx]

Supplementary Tables

The tables below provide details of the comparative accuracy testing of GROM and other state-of-the-art algorithms, selected as described in the Supplementary Methods, across an extensive number of the best available human variant benchmarks. See Fig. 1 for abbreviations and illustrations.

Supplementary Table 1. Algorithm comparison for NA12878 SNVs and indels. Comparison based on Illumina Platinum Pedigree benchmark (SNVs, n=3525488; deletion indels, n=256862; insertion indels, n=267162) and Illumina Platinum WGS dataset NA12878.GROM had highest sensitivity and precision, except for deletion indel precision. Bold text indicates highest value for each metric.

| **SNVs** | | | | |  |
| --- | --- | --- | --- | --- | --- |
| **Algorithm** | **True Positives** | **False Positives** | **Sensitivity (%)** | **Precision (%)** | |
| GATK-HC | 3496255 | 525285 | 99.17 | 86.94 | |
| SAMtools | 3488475 | 526514 | 98.95 | 86.89 | |
| GROM | 3497939 | 516965 | **99.22** | **87.12** | |
| **Deletion indels** | | | | | |
| **Algorithm** | **True Positives** | **FalsePositives** | **Sensitivity (%)** | **Precision (%)** | |
| GATK-HC | 238392 | 113136 | 92.81 | **67.82** | |
| SAMtools | 195945 | 149719 | 76.28 | 56.69 | |
| GROM | 239443 | 145762 | **93.22** | 62.16 | |
| **Insertion indels** | | | | |  |
| **Algorithm** | **True Positives** | **False Positives** | **Sensitivity (%)** | **Precision (%)** | |
| GATK-HC | 242152 | 112420 | 90.64 | 68.29 | |
| SAMtools | 204442 | 172777 | 76.52 | 54.20 | |
| GROM | 243187 | 112131 | **91.03** | **68.44** | |

Supplementary Table 2. Summary of SV deletion and duplication comparison. Comparison based on average ranking across benchmarks (1-highest to 3-lowest).Ranking for each benchmark based on sensitivity and precision values in Supplementary Tables 3-4. For instance, * Indicates tie.

| **Deletion (NA12878, Benchmark sets = 4)** | | |
| --- | --- | --- |
| **Algorithm** | **Sensitivity** | **Precision** |
| LUMPY | **1*** | 2 |
| Manta | 3 | 3 |
| GROM | **1*** | **1** |
| **Deletion (HX1, Benchmark sets = 3)** | | |
| **Algorithm** | **Sensitivity** | **Precision** |
| LUMPY | 2 | 3 |
| Manta | 3 | 2 |
| GROM | **1** | **1** |
| **Duplication (NA12878, Benchmark sets = 2)** | | |
| **Algorithm** | **Sensitivity** | **Precision** |
| LUMPY | 2 | 3 |
| Manta | 3 | **1** |
| GROM | **1** | 2 |
| **Duplication (HX1, Benchmark sets = 3)** | | |
| **Algorithm** | **Sensitivity** | **Precision** |
| LUMPY | 2 | 3 |
| Manta | 3 | 2 |
| GROM | **1** | **1** |

Supplementary Table 3. Algorithm comparison for NA12878 and HX1 deletions. Results for Illumina Platinum WGS datasets NA12878 and HX1. Bold text indicates highest value for each metric.

| **NA12878** | | | | |
| --- | --- | --- | --- | --- |
| **Algorithm** | **True Positives** | **False Positives** | **Sensitivity (%)** | **Precision (%)** |
| **DGV Gold Standard (truth set n=1930)** | | | | |
| LUMPY | 1213 | 3052 | 62.85 | 28.44 |
| Manta | 1138 | 3097 | 58.96 | 26.87 |
| GROM | 1237 | 2384 | **64.09** | **34.16** |
| **Mills Gold Standard (n=525)** | | | | |
| LUMPY | 332 | 3970 | 63.24 | 7.72 |
| Manta | 319 | 3914 | 60.76 | 7.54 |
| GROM | 363 | 3288 | **69.14** | **9.94** |
| **Genome in a Bottle (n=2676)** | | | | |
| LUMPY | 2338 | 1921 | **87.37** | 54.90 |
| Manta | 2235 | 1987 | 83.52 | 52.94 |
| GROM | 2269 | 1355 | 84.79 | **62.61** |
| **PacBio, Pendleton (n=7873)** | | | | |
| LUMPY | 2712 | 1635 | **34.45** | 62.39 |
| Manta | 2696 | 1550 | 34.24 | 63.50 |
| GROM | 2669 | 993 | 33.90 | **72.88** |
| **HX1** | | | | |
| **Algorithm** | **True Positives** | **False Positives** | **Sensitivity (%)** | **Precision (%)** |
| **DGV Gold Standard (truth set n=1552)** | | | | |
| LUMPY | 805 | 3727 | 51.87 | 17.76 |
| Manta | 749 | 2761 | 48.26 | 21.34 |
| GROM | 834 | 3021 | **53.74** | **21.63** |
| **PacBio, HX1 paper (n=9590)** | | | | |
| LUMPY | 2569 | 2238 | 26.79 | 53.44 |
| Manta | 3093 | 2084 | **32.25** | 59.75 |
| GROM | 2541 | 1674 | 26.50 | **60.28** |
| **IrysChip, HX1 paper (n=375)** | | | | |
| LUMPY | 211 | 3102 | 56.27 | 6.37 |
| Manta | 193 | 1875 | 51.47 | **9.33** |
| GROM | 212 | 2384 | **56.53** | 8.17 |

Supplementary Table 4. Algorithm comparison for NA12878 and HX1 duplications. Results for Illumina Platinum WGS datasets NA12878 and HX1. Bold text indicates highest value for each metric.

| **NA12878** | | | | |
| --- | --- | --- | --- | --- |
| **Algorithm** | **True Positives** | **False Positives** | **Sensitivity (%)** | **Precision (%)** |
| **DGV Gold Standard (truth set n=1707)** | | | | |
| LUMPY | 25 | 1081 | 1.46 | 2.26 |
| Manta | 9 | 185 | 0.53 | **4.64** |
| GROM | 54 | 1183 | **3.16** | 4.37 |
| **Mills Gold Standard (n=244)** | | | | |
| LUMPY | 10 | 1084 | 4.10 | 0.91 |
| Manta | 5 | 185 | 2.05 | **2.63** |
| GROM | 30 | 1189 | **12.30** | 2.46 |
| **HX1** | | | | |
| **Algorithm** | **True Positives** | **False Positives** | **Sensitivity (%)** | **Precision (%)** |
| **DGV Gold Standard (truth set n=342)** | | | | |
| LUMPY | 5 | 1145 | 1.46 | 0.43 |
| Manta | 0 | 197 | 0.00 | 0.00 |
| GROM | 13 | 752 | **3.80** | **1.70** |
| **PacBio, HX1 paper (n=9995)** | | | | |
| LUMPY | 75 | 1307 | 0.75 | 5.43 |
| Manta | 107 | 438 | 1.07 | **19.63** |
| GROM | 124 | 833 | **1.24** | 12.96 |
| **IrysChip, HX1 paper (n=776)** | | | | |
| LUMPY | 29 | 1850 | 3.74 | 1.54 |
| Manta | 12 | 386 | 1.55 | 3.02 |
| GROM | 38 | 1097 | **4.90** | **3.35** |

Supplementary Table 5. Algorithm comparison for NA12878 insertions. Results for Illumina Platinum dataset NA12878.Bold text indicates highest value for each metric.

| **Algorithm** | **True Positives** | **False Positives** | **Sensitivity (%)** | **Precision (%)** |
| --- | --- | --- | --- | --- |
| **Mills Gold Standard (truth set n=30)** | | | | |
| LUMPY | 0 | 0 | 0.00 | NA |
| Manta | 1 | 563 | 3.33 | 0.18 |
| GROM | 3 | 800 | **10.00** | **0.37** |
| **Genome in a Bottle (n=68)** | | | | |
| LUMPY | 0 | 0 | 0.00 | NA |
| Manta | 19 | 545 | 27.94 | 3.37 |
| GROM | 34 | 769 | **50.00** | **4.23** |

Supplementary Table 6. Algorithm comparison for NA12878 inversions. Comparison based on Pendleton PacBio benchmark (n=40) and Illumina Platinum WGS dataset NA12878. Bold text indicates highest value for each metric.

| **Algorithm** | **True Positives** | **False Positives** | **Sensitivity (%)** | **Precision (%)** |
| --- | --- | --- | --- | --- |
| LUMPY | 17 | 92 | 42.50 | 15.60 |
| Manta | 25 | 169 | 62.50 | 12.89 |
| GROM | 28 | 144 | **70.00** | **16.28** |

Supplementary Table 7. Run time comparison with and without duplicate filtering. SAMtools rmdup was chosen for duplicate filtering based on our duplicate filtering comparison (data not shown). GROM used GROM’s built-in duplicate filter. Bold text indicates the fastest algorithm for each genome/filtering combination.

| **Algorithm** | **Run Time (with/without duplicate filtering, minutes)** | |
| --- | --- | --- |
|  | **NA12878 (50x)** | **HX1 (68x)** |
| GATK-HC | 3021 / 2413 | 6139 / 5222 |
| SAMtools | 3946 / 3338 | 5485 / 4569 |
| LUMPY | 1255 / 647 | 1570 / 653 |
| Manta | 993 / 385 | 1433 / 517 |
| GROM | **211** / **222** | **235** / **248** |

Supplementary Table 8.Multithreading run time comparison with and without duplicate filtering. Sambamba was chosen for duplicate filtering due to its multithreading capability. GROM used its built-in duplicate filter. Run times measured on a 24-thread computer cluster. Optimal run time was achieved by dedicating 22 threads to GATK and 2 threads to Manta.

| **Algorithms** | **Multithreaded Run Time**  **(with/without duplicate filtering, minutes)** | |
| --- | --- | --- |
|  | **NA12878** | **HX1** |
| GATK-HC plus Manta | 794 / 684 | 1072/ 944 |
| GROM | 11 / 12 | 38/ 40 |

Supplementary Table 9. Validated GROM-specific SVs impacting genes. Validation score indicates how many validation benchmarks contained the variant (50% reciprocal overlap).These variants were not detected by LUMPY or Manta. Benchmarks: DGV-GS, Mills-GS, GIAB.

| **Location** | **Gene** | **SV Type** | **Validation Score** |
| --- | --- | --- | --- |
| 1:152760145-152770851 | LCE1D | deletion | 3 |
| 19:52133134-52150224 | SIGLEC5 | deletion | 3 |
| 1:25591191-25662106 | RHD | deletion | 2 |
| 1:110226443-110243018 | GSTM1 | deletion | 2 |
| 1:159015055-159019223 | IFI16 | deletion | 2 |
| 1:248725151-248798485 | OR2T34 | deletion | 2 |
| 3:75568629-75593356 | CTD-2026G6.2 | deletion | 2 |
| 11:4250161-4301187 | SSU72P5 | deletion | 2 |
| 11:4967800-4976245 | OR51A4 | deletion | 2 |
| 11:55365501-55458366 | OR4C11 | deletion | 2 |
| 12:11505044-11545313 | PRB1 | deletion | 2 |
| 17:18406914-18464818 | USP32P2 | deletion | 2 |
| 1:144954693-144955319 | PDE4DIP | deletion | 2 |
| 22:22987246-23247169 | GGTLC2 | deletion | 2 |
| 16:21507745-21594417 | CTD-2547E10.2 | duplication | 2 |
| 16:22617874-22710749 | RP11-105C19.1 | duplication | 2 |
| 16:32295808-32658086 | RP11-17M15.2 | duplication | 2 |
| 16:33293761-33633225 | RP11-23E10.5 | duplication | 2 |
| 16:70880975-71202571 | HYDIN | duplication | 2 |
| 17:16656414-16727134 | CCDC144A | duplication | 2 |
| 17:44165258-44369297 | KANSL1 | duplication | 2 |
| 19:54728433-54747094 | RPS9 | duplication | 2 |
| 4:69422464-69476891 | UGT2B17 | deletion | 1 |
| 7:38388448-38397626 | TRGV5 | deletion | 1 |
| 16:20544941-20550608 | ACSM2B | deletion | 1 |
| 16:75238619-75239036 | CTRB2 | deletion | 1 |
| 19:4511539-4511713 | PLIN4 | deletion | 1 |
| 1:17176016-17205499 | RP11-108M9.2 | duplication | 1 |
| 1:120532013-120637458 | NOTCH2 | duplication | 1 |
| 1:143910092-144074146 | FAM72D | duplication | 1 |
| 1:144672828-144710446 | WI2-1896O14.1 | duplication | 1 |
| 8:11979444-12008800 | USP17L7 | duplication | 1 |
| 15:94886479-94888503 | MCTP2 | duplication | 1 |

Supplementary Table 10. Example of duplication filtering methods comparison (for deletion indel detection by the respective pipelines) on Illumina WGS dataset NA12878. Performance evaluated with Illumina Platinum Pedigree validation set (n=256862).Bold text indicates highest value for each metric.

| **Duplicate Filter** | **True Positives** | **False Positives** | **Sensitivity (%)** | **Precision (%)** |
| --- | --- | --- | --- | --- |
| None | 239437 | 145812 | 93.216 | 62.151 |
| SAMtools | 239446 | 145800 | 93.220 | 62.154 |
| Sambamba | 239452 | 145932 | **93.222** | 62.133 |
| GROM | 239443 | 145762 | 93.219 | **62.160** |
