# Supplementary material for: Lightning-fast genome variant detection with GROM
Source: Gigascience. 2017 Sep 18;6(10):1–7. doi: 10.1093/gigascience/gix091 (PMC5737730; doi:10.1093/gigascience/gix091)
Supplement: Response-to-Reviewer-Comments_Original-Submission.pdf [file gix091_response-to-reviewer-comments_original-submission.pdf]

Author's Response To Reviewer Comments

See attached cover-response-reviewers file.
