# Supplementary material for: Lightning-fast genome variant detection with GROM
Source: Gigascience. 2017 Sep 18;6(10):1–7. doi: 10.1093/gigascience/gix091 (PMC5737730; doi:10.1093/gigascience/gix091)
Supplement: Response-to-Reviewer-Comments_Revision-1.pdf [file gix091_response-to-reviewer-comments_revision-1.pdf]

Author's Response To Reviewer Comments

Reviewer's comment:

1. With respect to the comment 6 I gave in my previous feedback, the authors are correct that there exists no validated translocation dataset for either NA12878 or HX1, so it's acceptable if the authors skip benchmarking the translocations detected by GROM by stating in the main text the reason why they have skipped benchmarking translocation, but not other variant types. However, the real problem is, by not reporting the number of translocations detected by GROM in the paper, the authors failed to provide any evidence to support the claim in abstract that GROM is capable to detect translocation.

Response:
We have added the following sentences to the Results to address this:

"GROM also detected 545 and 472 translocation events in the NA12878 and HX1, respectively. However, these events were not included in the benchmarking due to the lack of validated translocation datasets for either genome.

We have also modified the Abstract to indicate GROM's applicability to the datasets other than WGS, as previously requested.
