# Supplementary material for: Lightning-fast genome variant detection with GROM
Source: Gigascience. 2017 Sep 18;6(10):1–7. doi: 10.1093/gigascience/gix091 (PMC5737730; doi:10.1093/gigascience/gix091)
Supplement: Reviewer-1-Report-(Original-Submission).pdf [file gix091_reviewer-1-report-(original-submission).pdf]

Reviewer Report

Title: Lightning-fast genome variant detection with GROM

Version: Original Submission **Date:** 5/18/2017

Reviewer name: Ruibang Luo

Reviewer Comments to Author:

The manuscript titled "Lightning-fast genome variant detection with GROM" by Smith et al. presented a versatile variant caller that takes alignments as input and outputs identified SNPs, Indels, CNVs and SVs. The paper is well written. As reported, the sensitivity and precision of GROM ranked top by comparing to currently outperforming callers GATK-HaplotypeCaller and Manta. The source code is publically available, however, by the time this review is submitted, the existing version is still single-threaded (inferred by the caveat "DO NOT USE! PARALLEL VERSION BEING FINALIZED" in README, github commit 'cad40a8'). So we are unable to verify authors' claim that GROM is ~24 times faster than "GATK-HC + Manta".

Major concerns:
1. While an important contribution of GROM is its speed, and the experiments as reported are using 24 threads, the authors must provide a usable parallel version for benchmarking.
2. It took me a while to realize GROM doesn't perform alignment and works as a variant caller only. This confusion is confirmed by another colleague. Using the word "pipeline" in the abstract and citing SpeedSeq in the first paragraph of the introduction lead to this confusion, while pipeline usually refers to the whole secondary analysis, and SpeedSeq is a pipeline that performs alignment before variant calling. It would be best if the authors can make it clear as early as possible that GROM uses alignments as input and performs variant calling only.
3. GROM's applicability on Whole Exome Sequencing was not mentioned. While WES is still more popular and less expensive at this moment, GROM's applicability on WES should be a huge concern of readers.
4. GROM utilizes physical coverage, but it's unclear that how using libraries with multiple insert sizes will affect the result.

A few comments:
1. Page 2, line 28, in Abstract, ">50 times faster than pipelines detecting a similar range of variants" is an unsupported statement. Page 12, line 51, in Conclusion, "a minimum 24x faster than current state-of-the-art methods by incorporating comprehensive variant detection …" is contradicting the abstract.
2. Page 5, line 33, "is compared with lengths of existing clusters", cluster was not defined. It is difficult to see how a cluster is formed from individual reference bases.
3. Page 7, line 24. Supplementary table 10 is not referred. And the result of Picard is missing in Supplementary table 10.
4. How the two breakpoints of an inversion pair with each other is unclear to me.

5. Page 8, line 34, the version of GIAB used should be recorded.
6. GROM generates translocation by no translocations was included the results.
7. The hardware configuration for the benchmarks did not appear.

Ruibang Luo
Johns Hopkins University

Methods

Are the methods appropriate to the aims of the study, are they well described, and are necessary controls included? Yes

Conclusions

Are the conclusions adequately supported by the data shown? Yes

Reporting Standards

Does the manuscript adhere to the journal's guidelines on minimum standards of reporting? Yes Choose an item.

Statistics

Are you able to assess all statistics in the manuscript, including the appropriateness of statistical tests used? Yes, and I have assessed the statistics in my report.

Quality of Written English

Please indicate the quality of language in the manuscript: Acceptable

Declaration of Competing Interests

Please complete a declaration of competing interests, considering the following questions:

- Have you in the past five years received reimbursements, fees, funding, or salary from an organisation that may in any way gain or lose financially from the publication of this manuscript, either now or in the future?
- Do you hold any stocks or shares in an organisation that may in any way gain or lose financially from the publication of this manuscript, either now or in the future?
- Do you hold or are you currently applying for any patents relating to the content of the manuscript?
- Have you received reimbursements, fees, funding, or salary from an organization that holds or has applied for patents relating to the content of the manuscript?
- Do you have any other financial competing interests?

- Do you have any non-financial competing interests in relation to this paper?

If you can answer no to all of the above, write 'I declare that I have no competing interests' below. If your reply is yes to any, please give details below.

I declare that I have no competing interests

I agree to the open peer review policy of the journal. I understand that my name will be included on my report to the authors and, if the manuscript is accepted for publication, my named report including any attachments I upload will be posted on the website along with the authors' responses. I agree for my report to be made available under an Open Access Creative Commons CC-BY license (http://creativecommons.org/licenses/by/4.0/). I understand that any comments which I do not wish to be included in my named report can be included as confidential comments to the editors, which will not be published.

I agree to the open peer review policy of the journal

To further support our reviewers, we have joined with Publons, where you can gain additional credit to further highlight your hard work (see: https://publons.com/journal/530/gigascience). On publication of this paper, your review will be automatically added to Publons, you can then choose whether or not to claim your Publons credit. I understand this statement.

Yes
