# Supplementary material for: Lightning-fast genome variant detection with GROM
Source: Gigascience. 2017 Sep 18;6(10):1–7. doi: 10.1093/gigascience/gix091 (PMC5737730; doi:10.1093/gigascience/gix091)
Supplement: Reviewer-2-Report-(Original-Submission).pdf [file gix091_reviewer-2-report-(original-submission).pdf]

Reviewer Report

Title: Lightning-fast genome variant detection with GROM

Version: Original Submission **Date:** 6/9/2017

Reviewer name: Suzanne Sindi

Reviewer Comments to Author:

The authors present a rapid integrative code base for predicting a variety of genomic variants (SNVs, SVs) from mapped sequencing data. While having an integrated platform is useful and their work is sound, I have the following questions which I believe would improve the manuscript and lend clarity as to the novelty of the work.

Questions:

(1) A major source of motivation for the method states that "computational analysis…often takes months of longer to complete". There is no citation or justification of where the computational time and cost is spent.

I have run several SV only detection method on high coverage genomes and it typically does not take more than a day with the computational bottleneck being the input/reading of the BAM files.

I think it is more than sufficient to say that your code base offers what appears to be around a 20-fold speed-up over competing approaches. That in itself seems to be the key point while this "months" statement seems overkill.

(2) The algorithm requires a BAM file but - what seems to be missing from the manuscript - is that this BAM file seems to need to be sorted by location. Depending on the size of the BAM file, sorting can take an extremely long time. There are variant detection methods (at least for SVs) which do not require a sorted BAM file.

Assuming I'm correct that your work requires sorted BAM files, can you comment on how this changes the computational time?

(3) Numbering your equations - and referring to them appropriately - would improve the readability of your manuscript.

In addition, in the equation which defines breakpoint evidence for a cluster - you include the number of previously recorded reads. This was confusing to me, does that mean the number of presently recorded reads - in support of a cluster - influences whether or not a read is added to the cluster?

Finally, again at least for SVs, there are several manuscripts that describe when reads support the same structural variant. For example,

- Hormozdiari, Fereydoun, et al. "Combinatorial algorithms for structural variation detection in high-throughput sequenced genomes." Genome research 19.7 (2009): 1270-1278.
- Sindi, Suzanne, et al. "A geometric approach for classification and comparison of structural variants." Bioinformatics 25.12 (2009): i222-i230.

It is not clear to me if your clustering method is any different from these and - if it is different - what advantage/disadvantages your method poses.

(4) While your algorithm has faster run-time, it is not clear what the memory costs are.

For example, in cancer genomes where there are variants of many kinds - performing all the clustering "on the fly" while the BAM file is being processed would be prohibitive because of the memory usage associated.

(5) Finally, I believe precision/recall information should be given in the main manuscript and not the supplement. Given the high (though decreasing) costs of DNA sequencing and the significant health impact even a 20-times speed-up is not meaningful unless the results are more accurate.

Methods

Are the methods appropriate to the aims of the study, are they well described, and are necessary controls included? Yes

Conclusions

Are the conclusions adequately supported by the data shown? Yes

Reporting Standards

Does the manuscript adhere to the journal's guidelines on [minimum standards of reporting?]() Yes Choose an item.

Statistics

Are you able to assess all statistics in the manuscript, including the appropriateness of statistical tests used? Yes, and I have assessed the statistics in my report.

Quality of Written English

Please indicate the quality of language in the manuscript: Acceptable

Declaration of Competing Interests

Please complete a declaration of competing interests, considering the following questions:

- Have you in the past five years received reimbursements, fees, funding, or salary from an organisation that may in any way gain or lose financially from the publication of this manuscript, either now or in the future?
- Do you hold any stocks or shares in an organisation that may in any way gain or lose financially from the publication of this manuscript, either now or in the future?
- Do you hold or are you currently applying for any patents relating to the content of the manuscript?
- Have you received reimbursements, fees, funding, or salary from an organization that holds or has applied for patents relating to the content of the manuscript?
- Do you have any other financial competing interests?
- Do you have any non-financial competing interests in relation to this paper?

If you can answer no to all of the above, write 'I declare that I have no competing interests' below. If your reply is yes to any, please give details below.

I declare that I have no competing interests

I agree to the open peer review policy of the journal. I understand that my name will be included on my report to the authors and, if the manuscript is accepted for publication, my named report including any attachments I upload will be posted on the website along with the authors' responses. I agree for my report to be made available under an Open Access Creative Commons CC-BY license (http://creativecommons.org/licenses/by/4.0/). I understand that any comments which I do not wish to be included in my named report can be included as confidential comments to the editors, which will not be published.

I agree to the open peer review policy of the journal

To further support our reviewers, we have joined with Publons, where you can gain additional credit to further highlight your hard work (see: https://publons.com/journal/530/gigascience). On publication of this paper, your review will be automatically added to Publons, you can then choose whether or not to claim your Publons credit. I understand this statement.

Yes
